# Supplementary material for: Optimizing Genetic Workup in Pheochromocytoma and Paraganglioma by Integrating Diagnostic and Research Approaches
Source: Cancers (Basel). 2019 Jun 11;11(6):809. doi: 10.3390/cancers11060809 (PMC6627084; doi:10.3390/cancers11060809)
Supplement: Supplementary file 1 [file cancers-11-00809-s001.zip › Supplementary_files/Supplementary_Figure_S1.pdf]

Supplementary Figure S1: CNV plots

CNV plots of analyzed samples and controls . For every analyzed gene, log2 ratios in comparison to ten normal controls were plotted for every sample.

Article

Optimizing Genetic Workup in Pheochromocytoma and Paraganglioma by Integrating Diagnostic and Research Approaches

Laura Gieldon[1, 2,#], Doreen William[1,#], Karl Hackmann[1,2], Winnie Jahn[1], Arne Jahn[1,2], Johannes Wagner[1,2], Andreas Rump[1,2], Nicole Bechmann[3], Svenja Nölting[4], Thomas Knösel[5], Volker Gudziol[6], Georgiana Constantinescu[7], Jimmy Masjkur[7], Felix Beuschlein[8], Henri JLM Timmers[9], Letizia Canu[10], Karel Pacak[11], Mercedes Robledo[12], Daniela Aust[13], Evelin Schröck[1,2], Graeme Eisenhofer[3,7], Susan Richter[3] and Barbara Klink[1,2,14,*]

ID1

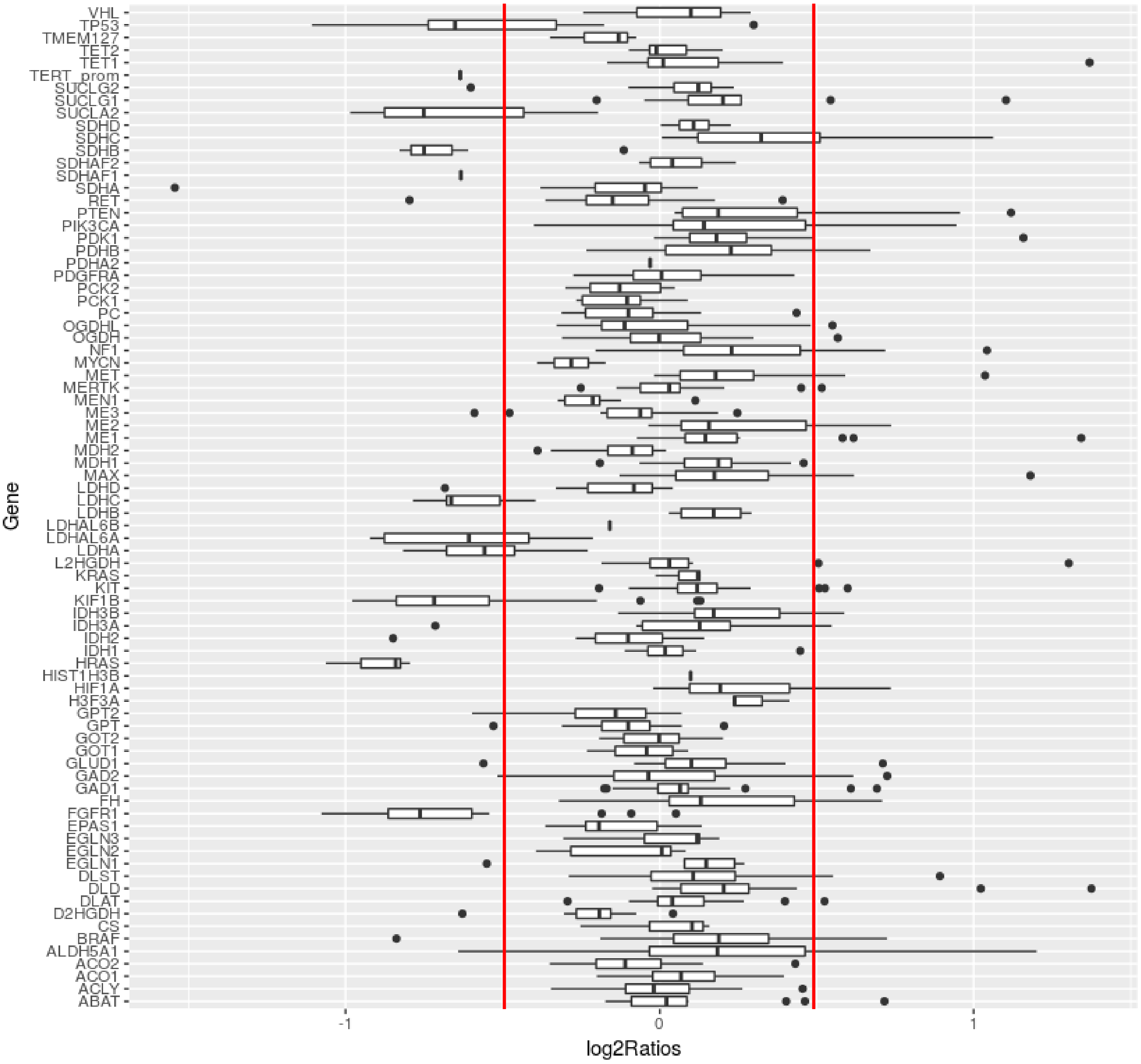

VHL
TP53
TMEM127
TET2
TET1
TERT_prom
SUCLG2
SUCLG1
SUCLA2
SDHD
SDHC
SDHB
SDHAF2
SDHAF1
SDHA
RET
PTEN
PIK3CA
PDK1
PDHB
PDHA2
PDGFRA
PCK2
PCK1
PC
OGDHL
OGDH
NF1
MYCN
MET
MERTK
MEN1
ME3
ME2
ME1
MDH2
MDH1
MAX
LDHD
LDHC
LDHB
LDHAL6B
LDHAL6A
LDHA
L2HGDH
KRAS
KIT
KIF1B
IDH3B
IDH3A
IDH2
IDH1
HRAS
HIST1H3B
HIF1A
H3F3A
GPT2
GPT
GOT2
GOT1
GLUD1
GAD2
GAD1
FH
FGFR1
EPAS1
EGLN3
EGLN2
EGLN1
DLST
DLD
DLAT
D2HGDH
CS
BRAF
ALDH5A1
ACO2
ACO1
ACLY
ABAT
Gene
-1
0
1
log2Ratios

ID24

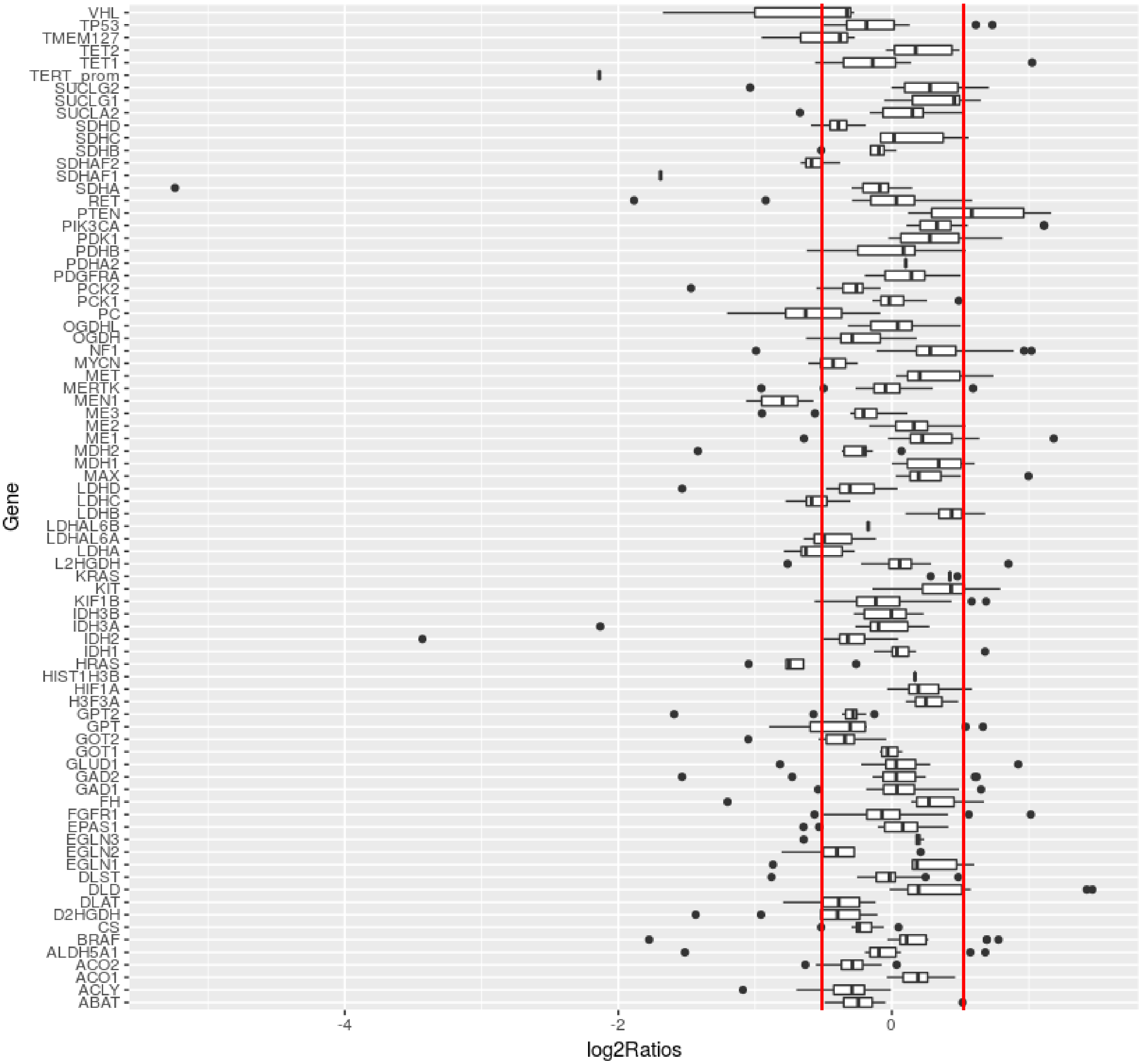

Gene
log2Ratios
-4
-2
0
VHL
TP53
TMEM127
TET2
TET1
TERT_prom
SUCLG2
SUCLG1
SUCLA2
SDHD
SDHC
SDHB
SDHAF2
SDHAF1
SDHA
RET
PTEN
PIK3CA
PDK1
PDHB
PDHA2
PDGFRA
PCK2
PCK1
PC
OGDHL
OGDH
NF1
MYCN
MET
MERTK
MEN1
ME3
ME2
ME1
MDH2
MDH1
MAX
LDHD
LDHC
LDHB
LDHAL6B
LDHAL6A
LDHA
L2HGDH
KRAS
KIT
KIF1B
IDH3B
IDH3A
IDH2
IDH1
HRAS
HIST1H3B
HIF1A
H3F3A
GPT2
GPT
GOT2
GOT1
GLUD1
GAD2
GAD1
FH
FGFR1
EPAS1
EGLN3
EGLN2
EGLN1
DLST
DLD
DLAT
D2HGDH
CS
BRAF
ALDH5A1
ACO2
ACO1
ACLY
ABAT

ID41

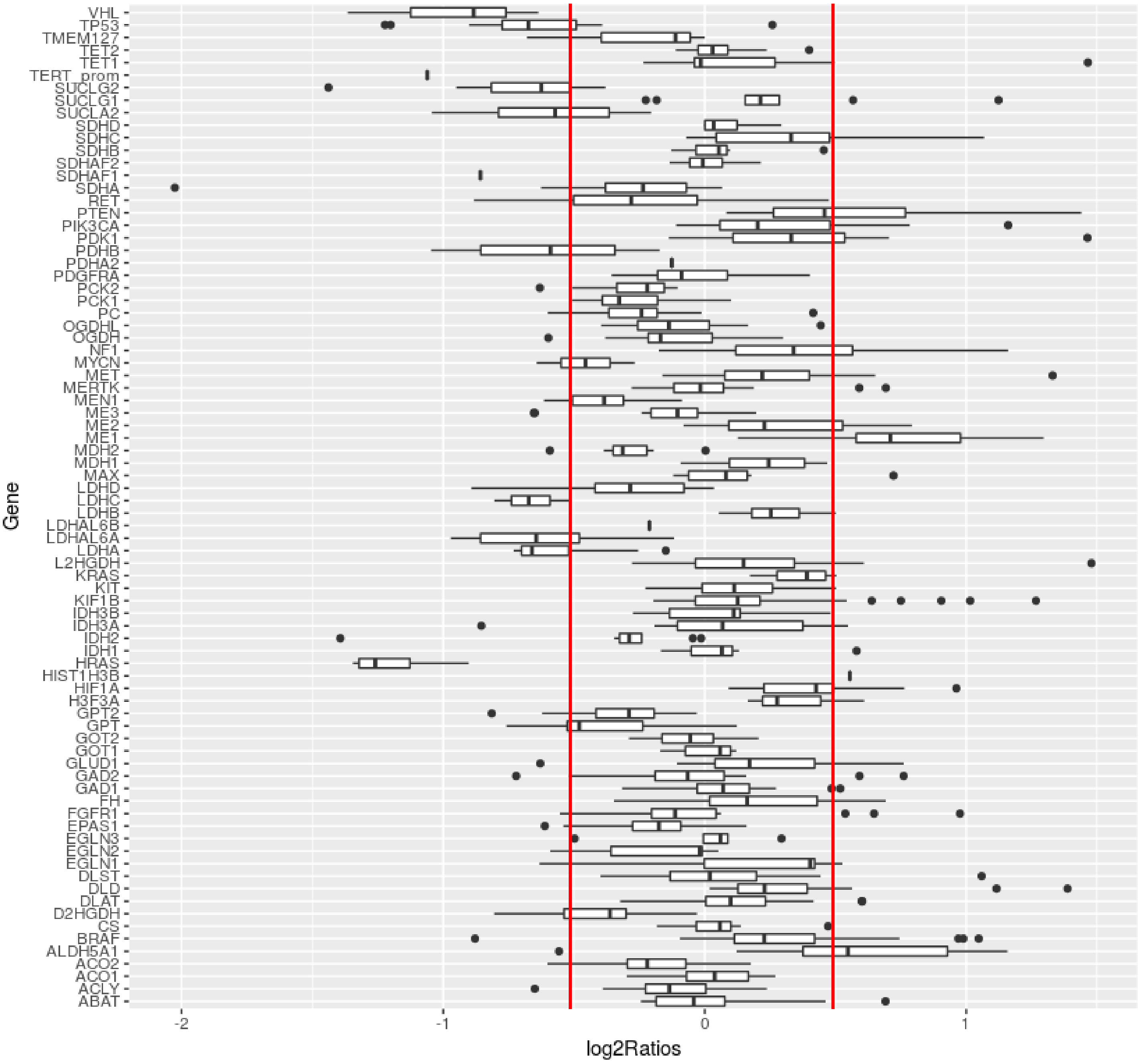

Gene
log2Ratios
-2
-1
0
1
VHL
TP53
TMEM127
TET2
TET1
TERT_prom
SUCLG2
SUCLG1
SUCLA2
SDHD
SDHC
SDHB
SDHAF2
SDHAF1
SDHA
RET
PTEN
PIK3CA
PDK1
PDHB
PDHA2
PDGFRA
PCK2
PCK1
PC
OGDHL
OGDH
NF1
MYCN
MET
MERTK
MEN1
ME3
ME2
ME1
MDH2
MDH1
MAX
LDHD
LDHC
LDHB
LDHAL6B
LDHAL6A
LDHA
L2HGDH
KRAS
KIT
KIF1B
IDH3B
IDH3A
IDH2
IDH1
HRAS
HIST1H3B
HIF1A
H3F3A
GPT2
GPT
GOT2
GOT1
GLUD1
GAD2
GAD1
FH
FGFR1
EPAS1
EGLN3
EGLN2
EGLN1
DLST
DLD
DLAT
D2HGDH
CS
BRAF
ALDH5A1
ACO2
ACO1
ACLY
ABAT

ID42

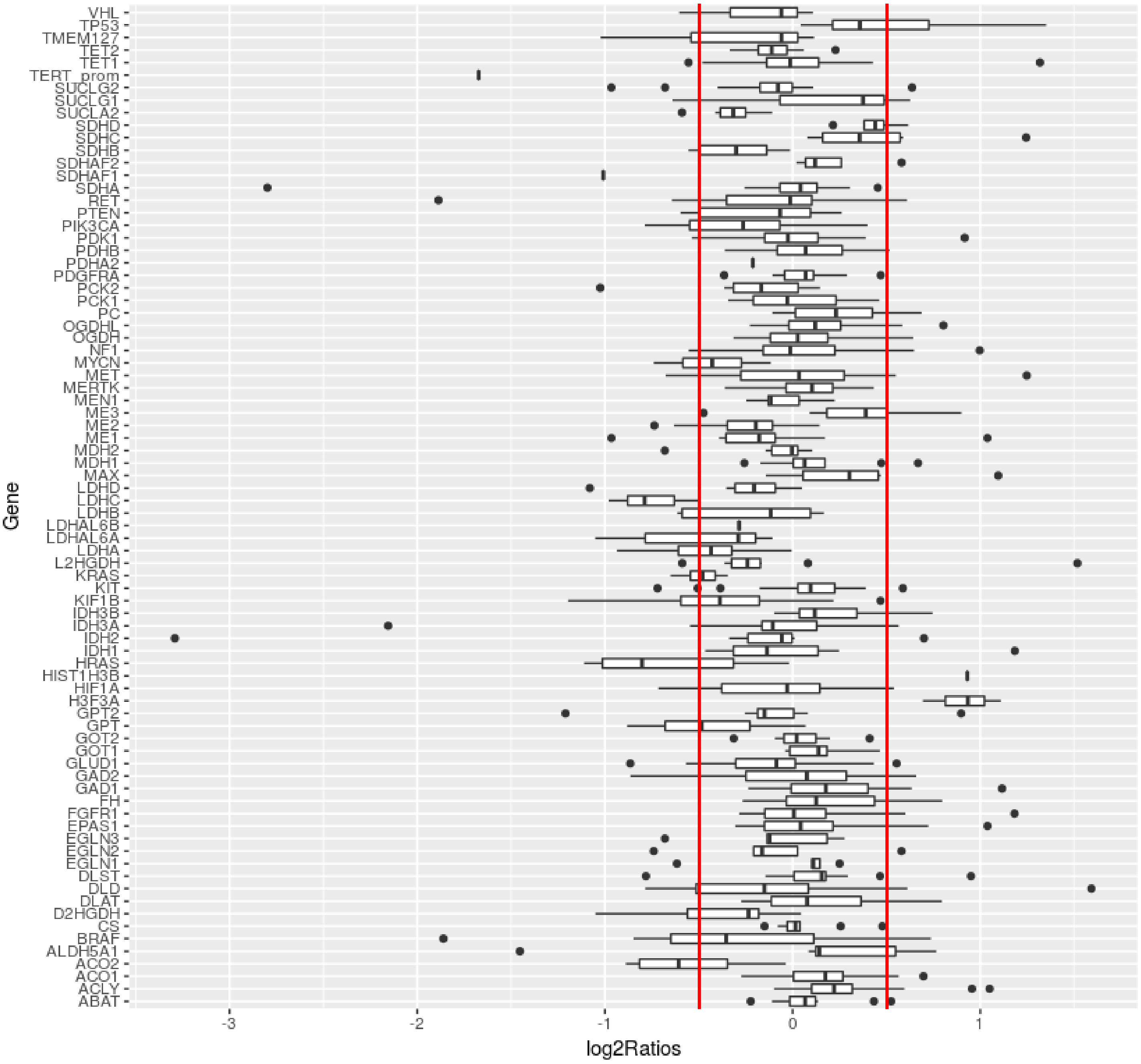

VHL
TP53
TMEM127
TET2
TET1
TERT_prom
SUCLG2
SUCLG1
SUCLA2
SDHD
SDHC
SDHB
SDHAF2
SDHAF1
SDHA
RET
PTEN
PIK3CA
PDK1
PDHB
PDHA2
PDGFRA
PCK2
PCK1
PC
OGDHL
OGDH
NF1
MYCN
MET
MERTK
MEN1
ME3
ME2
ME1
MDH2
MDH1
MAX
LDHD
LDHC
LDHB
LDHAL6B
LDHAL6A
LDHA
L2HGDH
KRAS
KIT
KIF1B
IDH3B
IDH3A
IDH2
IDH1
HRAS
HIST1H3B
HIF1A
H3F3A
GPT2
GPT
GOT2
GOT1
GLUD1
GAD2
GAD1
FH
FGFR1
EPAS1
EGLN3
EGLN2
EGLN1
DLST
DLD
DLAT
D2HGDH
CS
BRAF
ALDH5A1
ACO2
ACO1
ACLY
ABAT
Gene
-3
-2
-1
0
1
log2Ratios

ID43

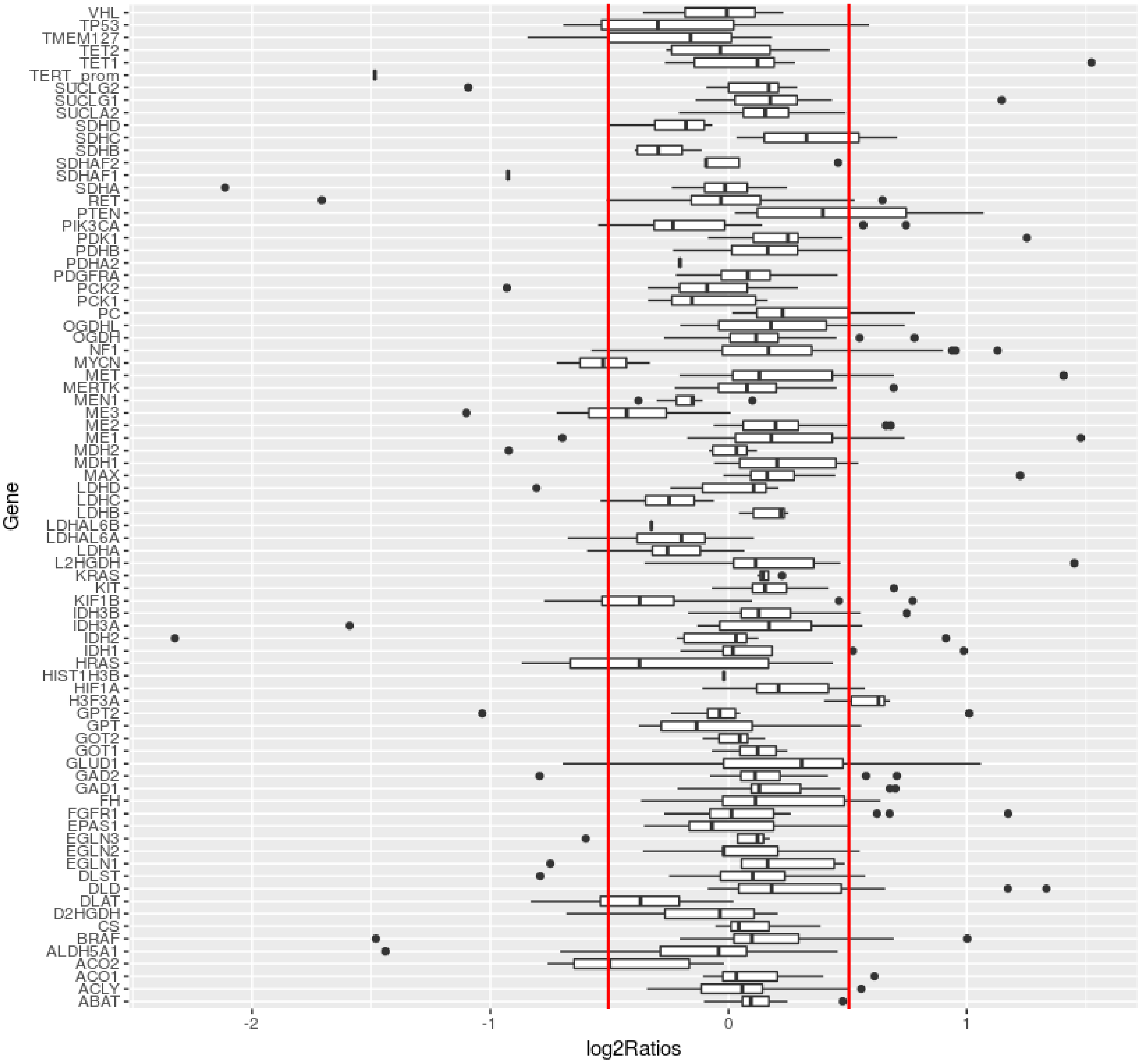
VHL
TP53
TMEM127
TET2
TET1
TERT_prom
SUCLG2
SUCLG1
SUCLA2
SDHD
SDHC
SDHB
SDHAF2
SDHAF1
SDHA
RET
PTEN
PIK3CA
PDK1
PDHB
PDHA2
PDGFRA
PCK2
PCK1
PC
OGDHL
OGDH
NF1
MYCN
MET
MERTK
MEN1
ME3
ME2
ME1
MDH2
MDH1
MAX
LDHD
LDHC
LDHB
LDHAL6B
LDHAL6A
LDHA
L2HGDH
KRAS
KIT
KIF1B
IDH3B
IDH3A
IDH2
IDH1
HRAS
HIST1H3B
HIF1A
H3F3A
GPT2
GPT
GOT2
GOT1
GLUD1
GAD2
GAD1
FH
FGFR1
EPAS1
EGLN3
EGLN2
EGLN1
DLST
DLD
DLAT
D2HGDH
CS
BRAF
ALDH5A1
ACO2
ACO1
ACLY
ABAT
Gene
-2
-1
0
1
log2Ratios

ID49

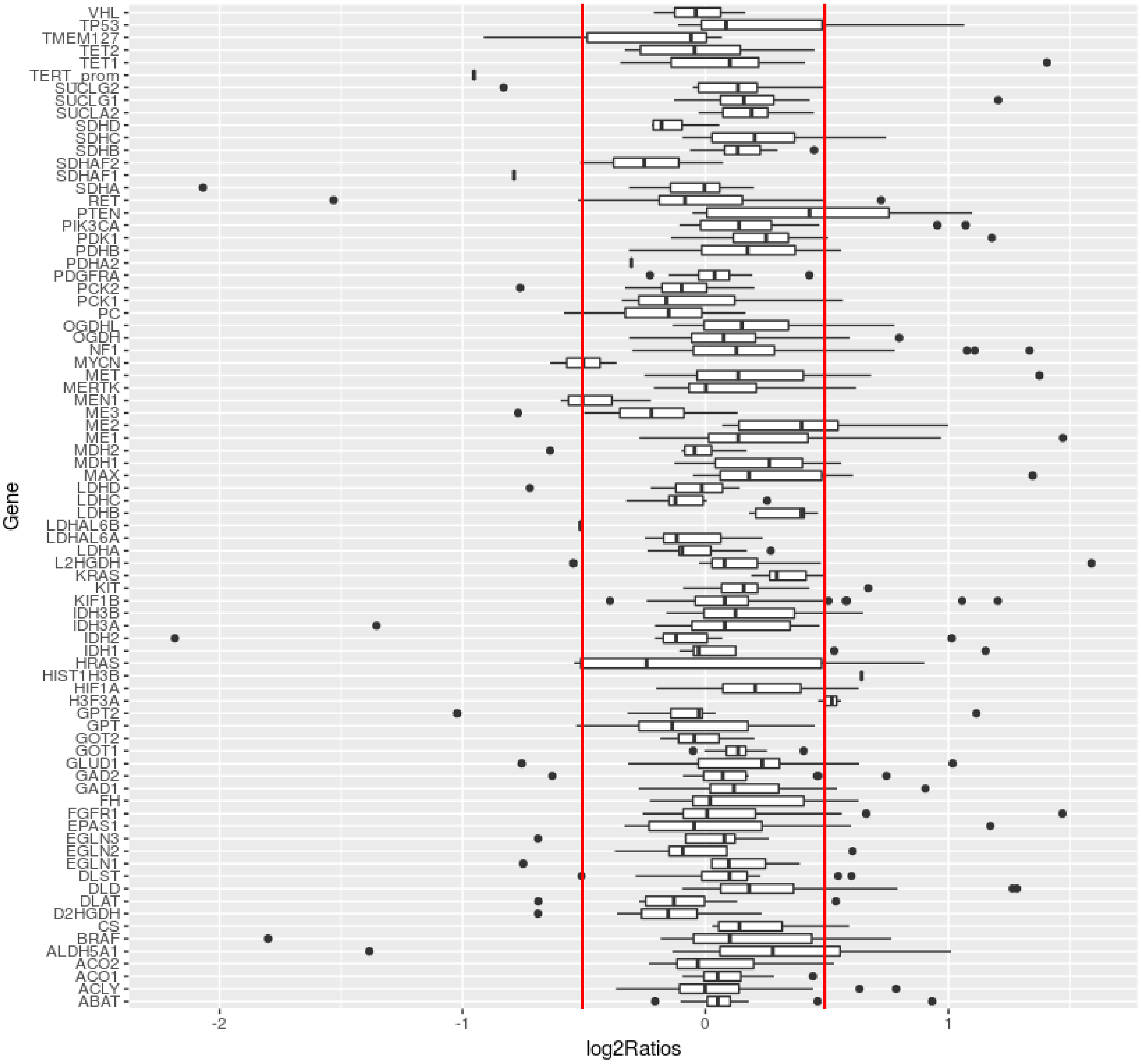

VHL
TP53
TMEM127
TET2
TET1
TERT_prom
SUCLG2
SUCLG1
SUCLA2
SDHD
SDHC
SDHB
SDHAF2
SDHAF1
SDHA
RET
PTEN
PIK3CA
PDK1
PDHB
PDHA2
PDGFRA
PCK2
PCK1
PC
OGDHL
OGDH
NF1
MYCN
MET
MERTK
MEN1
ME3
ME2
ME1
MDH2
MDH1
MAX
LDHD
LDHC
LDHB
LDHAL6B
LDHAL6A
LDHA
L2HGDH
KRAS
KIT
KIF1B
IDH3B
IDH3A
IDH2
IDH1
HRAS
HIST1H3B
HIF1A
H3F3A
GPT2
GPT
GOT2
GOT1
GLUD1
GAD2
GAD1
FH
FGFR1
EPAS1
EGLN3
EGLN2
EGLN1
DLST
DLD
DLAT
D2HGDH
CS
BRAF
ALDH5A1
ACO2
ACO1
ACLY
ABAT
Gene
-2
-1
0
1
log2Ratios

ID51

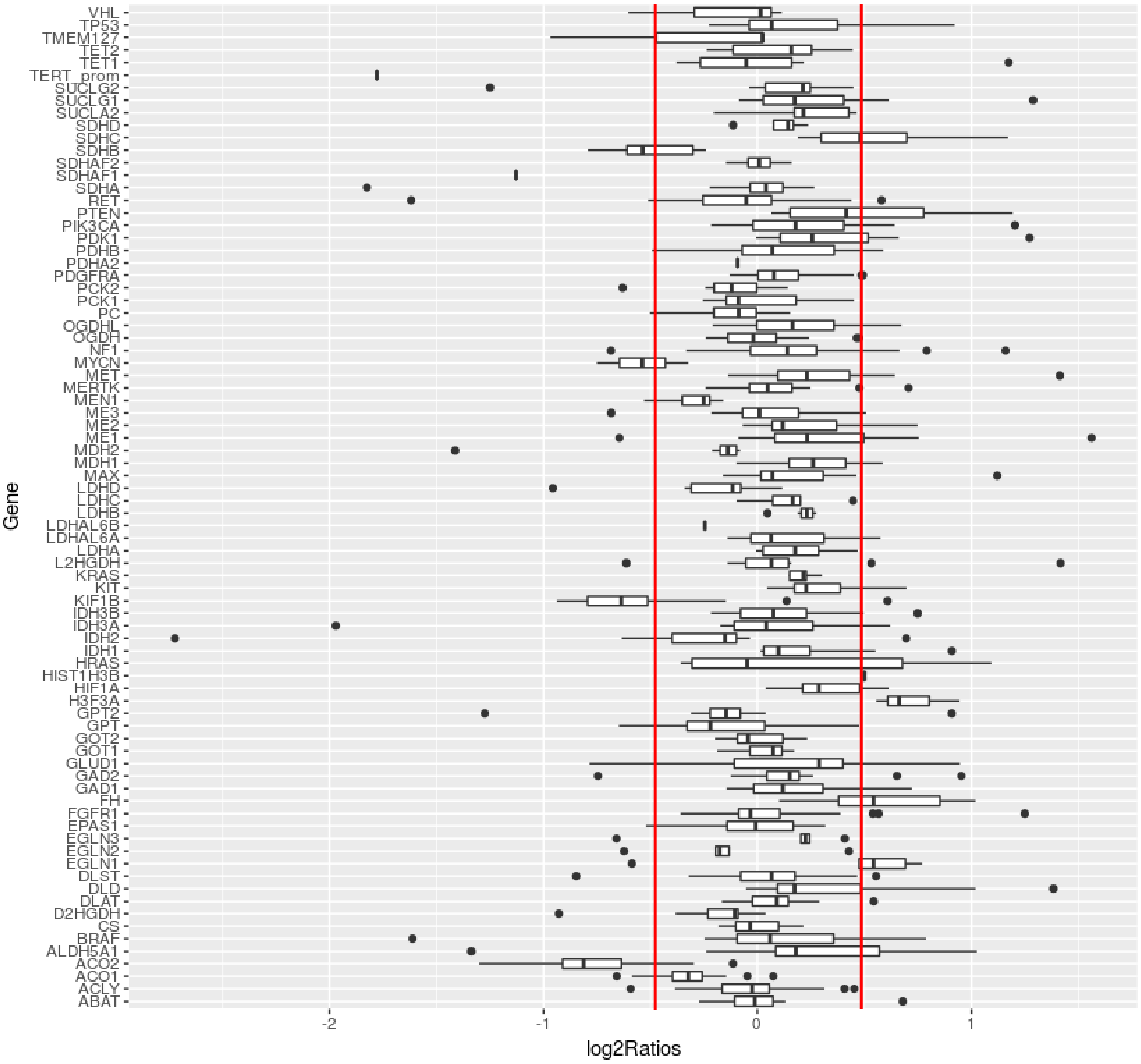
VHL
TP53
TMEM127
TET2
TET1
TERT_prom
SUCLG2
SUCLG1
SUCLA2
SDHD
SDHC
SDHB
SDHAF2
SDHAF1
SDHA
RET
PTEN
PIK3CA
PDK1
PDHB
PDHA2
PDGFRA
PCK2
PCK1
PC
OGDHL
OGDH
NF1
MYCN
MET
MERTK
MEN1
ME3
ME2
ME1
MDH2
MDH1
MAX
LDHD
LDHC
LDHB
LDHAL6B
LDHAL6A
LDHA
L2HGDH
KRAS
KIT
KIF1B
IDH3B
IDH3A
IDH2
IDH1
HRAS
HIST1H3B
HIF1A
H3F3A
GPT2
GPT
GOT2
GOT1
GLUD1
GAD2
GAD1
FH
FGFR1
EPAS1
EGLN3
EGLN2
EGLN1
DLST
DLD
DLAT
D2HGDH
CS
BRAF
ALDH5A1
ACO2
ACO1
ACLY
ABAT
Gene
-2
-1
0
1
log2Ratios

ID60

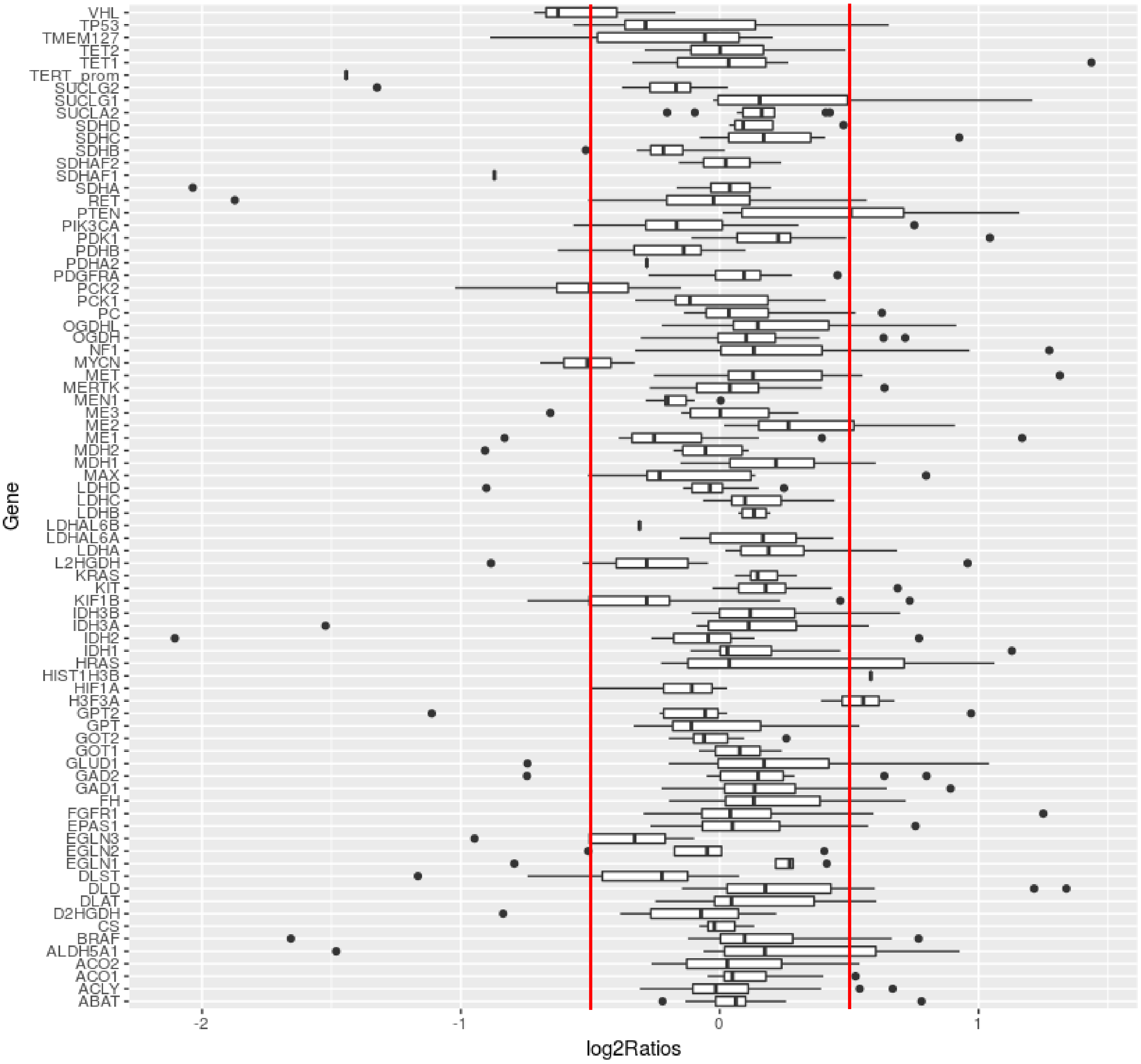
VHL
TP53
TMEM127
TET2
TET1
TERT_prom
SUCLG2
SUCLG1
SUCLA2
SDHD
SDHC
SDHB
SDHAF2
SDHAF1
SDHA
RET
PTEN
PIK3CA
PDK1
PDHB
PDHA2
PDGFRA
PCK2
PCK1
PC
OGDHL
OGDH
NF1
MYCN
MET
MERTK
MEN1
ME3
ME2
ME1
MDH2
MDH1
MAX
LDHD
LDHC
LDHB
LDHAL6B
LDHAL6A
LDHA
L2HGDH
KRAS
KIT
KIF1B
IDH3B
IDH3A
IDH2
IDH1
HRAS
HIST1H3B
HIF1A
H3F3A
GPT2
GPT
GOT2
GOT1
GLUD1
GAD2
GAD1
FH
FGFR1
EPAS1
EGLN3
EGLN2
EGLN1
DLST
DLD
DLAT
D2HGDH
CS
BRAF
ALDH5A1
ACO2
ACO1
ACLY
ABAT
Gene
-2
-1
0
1
log2Ratios

ID61

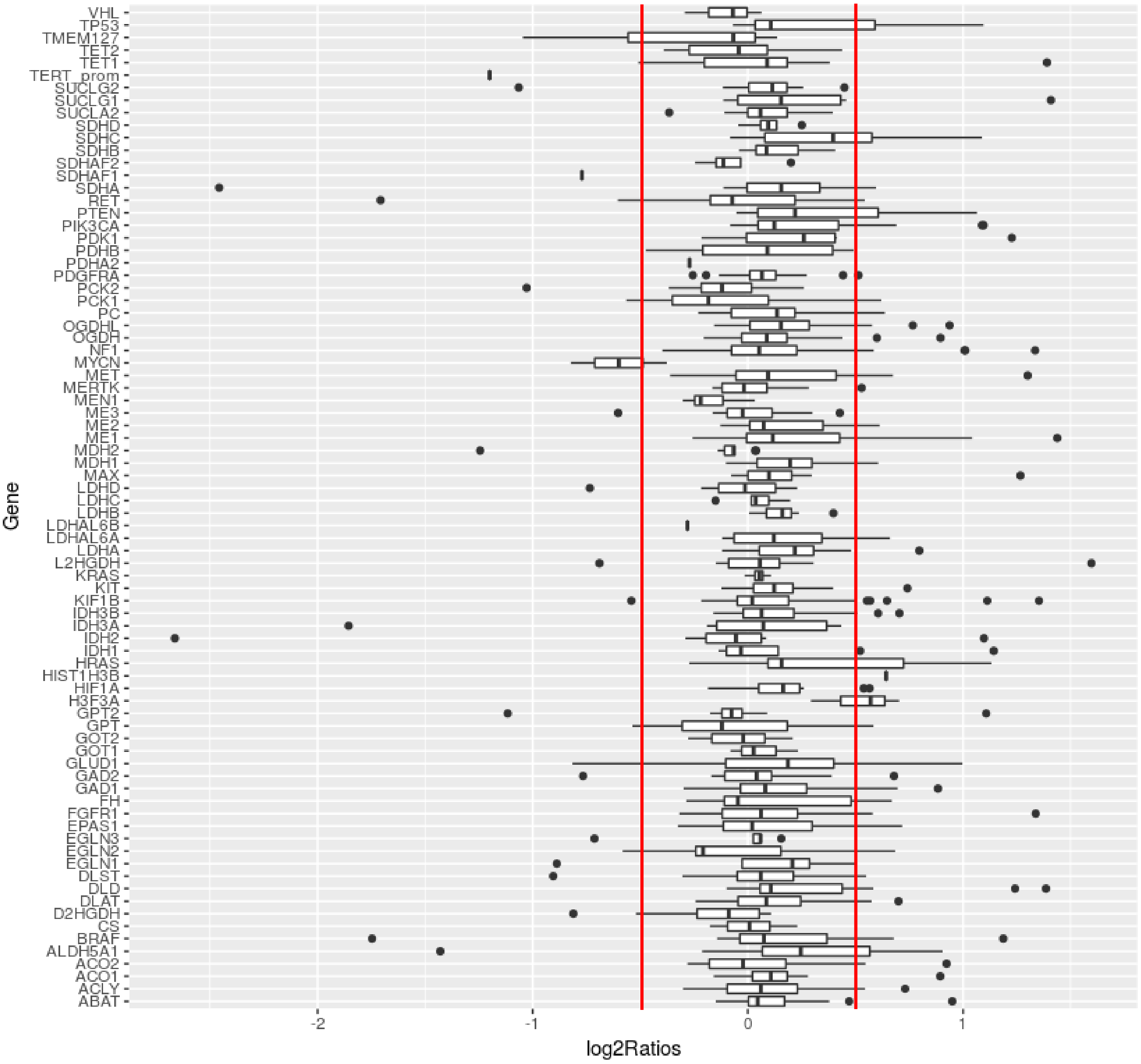

VHL
TP53
TMEM127
TET2
TET1
TERT_prom
SUCLG2
SUCLG1
SUCLA2
SDHD
SDHC
SDHB
SDHAF2
SDHAF1
SDHA
RET
PTEN
PIK3CA
PDK1
PDHB
PDHA2
PDGFRA
PCK2
PCK1
PC
OGDHL
OGDH
NF1
MYCN
MET
MERTK
MEN1
ME3
ME2
ME1
MDH2
MDH1
MAX
LDHD
LDHC
LDHB
LDHAL6B
LDHAL6A
LDHA
L2HGDH
KRAS
KIT
KIF1B
IDH3B
IDH3A
IDH2
IDH1
HRAS
HIST1H3B
HIF1A
H3F3A
GPT2
GPT
GOT2
GOT1
GLUD1
GAD2
GAD1
FH
FGFR1
EPAS1
EGLN3
EGLN2
EGLN1
DLST
DLD
DLAT
D2HGDH
CS
BRAF
ALDH5A1
ACO2
ACO1
ACLY
ABAT
Gene
-2
-1
0
1
log2Ratios

ID66

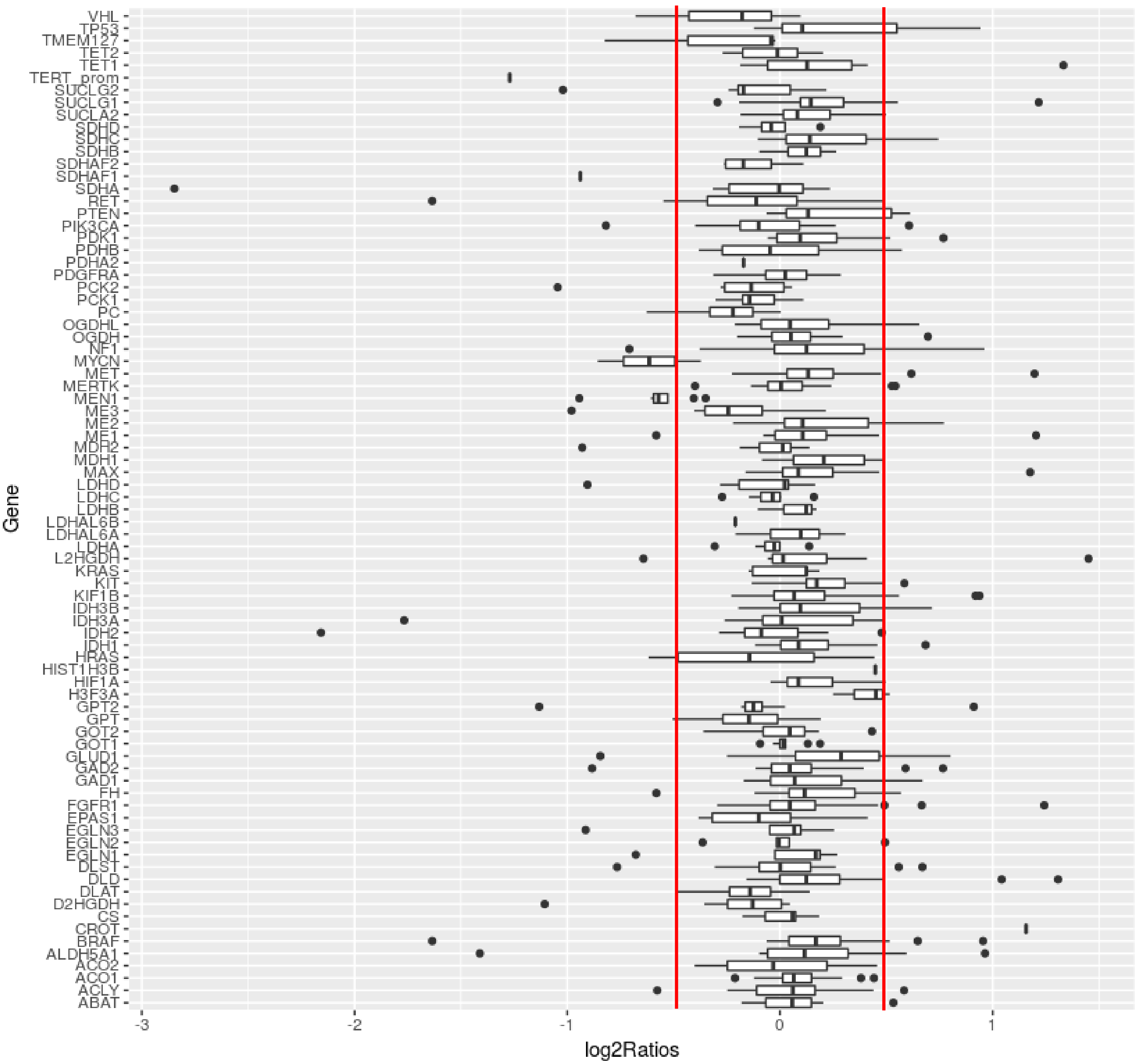

VHL
TP53
TMEM127
TET2
TET1
TERT_prom
SUCLG2
SUCLG1
SUCLA2
SDHD
SDHC
SDHB
SDHAF2
SDHAF1
SDHA
RET
PTEN
PIK3CA
PDK1
PDHB
PDHA2
PDGFRA
PCK2
PCK1
PC
OGDHL
OGDH
NF1
MYCN
MET
MERTK
MEN1
ME3
ME2
ME1
MDH2
MDH1
MAX
LDHD
LDHC
LDHB
LDHAL6B
LDHAL6A
LDHA
L2HGDH
KRAS
KIT
KIF1B
IDH3B
IDH3A
IDH2
IDH1
HRAS
HIST1H3B
HIF1A
H3F3A
GPT2
GPT
GOT2
GOT1
GLUD1
GAD2
GAD1
FH
FGFR1
EPAS1
EGLN3
EGLN2
EGLN1
DLST
DLD
DLAT
D2HGDH
CS
CROT
BRAF
ALDH5A1
ACO2
ACO1
ACLY
ABAT
Gene
-3
-2
-1
0
1
log2Ratios

ID67

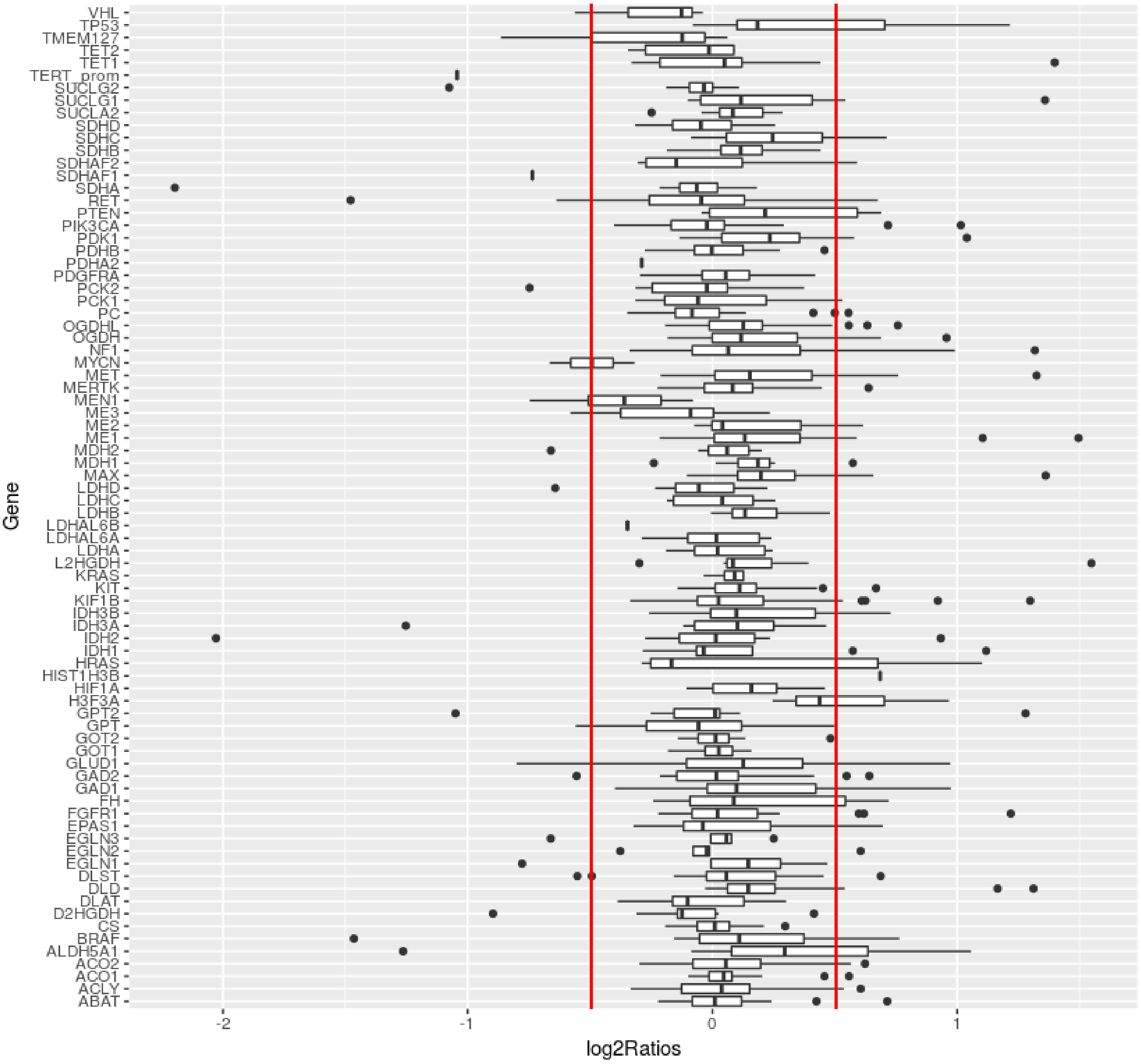

VHL
TP53
TMEM127
TET2
TET1
TERT_prom
SUCLG2
SUCLG1
SUCLA2
SDHD
SDHC
SDHB
SDHAF2
SDHAF1
SDHA
RET
PTEN
PIK3CA
PDK1
PDHB
PDHA2
PDGFRA
PCK2
PCK1
PC
OGDHL
OGDH
NF1
MYCN
MET
MERTK
MEN1
ME3
ME2
ME1
MDH2
MDH1
MAX
LDHD
LDHC
LDHB
LDHAL6B
LDHAL6A
LDHA
L2HGDH
KRAS
KIT
KIF1B
IDH3B
IDH3A
IDH2
IDH1
HRAS
HIST1H3B
HIF1A
H3F3A
GPT2
GPT
GOT2
GOT1
GLUD1
GAD2
GAD1
FH
FGFR1
EPAS1
EGLN3
EGLN2
EGLN1
DLST
DLD
DLAT
D2HGDH
CS
BRAF
ALDH5A1
ACO2
ACO1
ACLY
ABAT
Gene
-2
-1
0
1
log2Ratios

ID68

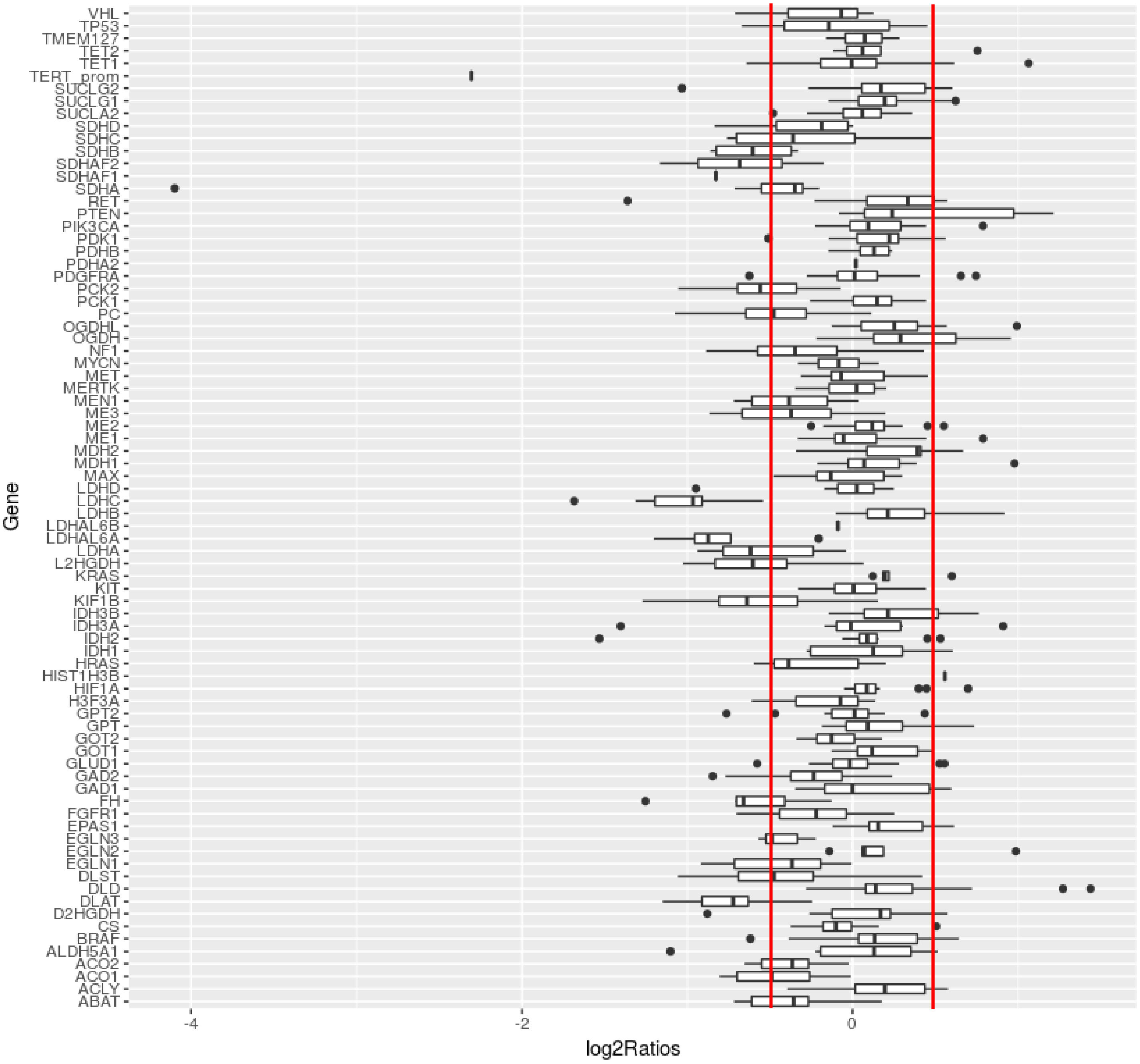
VHL
TP53
TMEM127
TET2
TET1
TERT_prom
SUCLG2
SUCLG1
SUCLA2
SDHD
SDHC
SDHB
SDHAF2
SDHAF1
SDHA
RET
PTEN
PIK3CA
PDK1
PDHB
PDHA2
PDGFRA
PCK2
PCK1
PC
OGDHL
OGDH
NF1
MYCN
MET
MERTK
MEN1
ME3
ME2
ME1
MDH2
MDH1
MAX
LDHD
LDHC
LDHB
LDHAL6B
LDHAL6A
LDHA
L2HGDH
KRAS
KIT
KIF1B
IDH3B
IDH3A
IDH2
IDH1
HRAS
HIST1H3B
HIF1A
H3F3A
GPT2
GPT
GOT2
GOT1
GLUD1
GAD2
GAD1
FH
FGFR1
EPAS1
EGLN3
EGLN2
EGLN1
DLST
DLD
DLAT
D2HGDH
CS
BRAF
ALDH5A1
ACO2
ACO1
ACLY
ABAT
Gene
-4
-2
0
log2Ratios

ID69

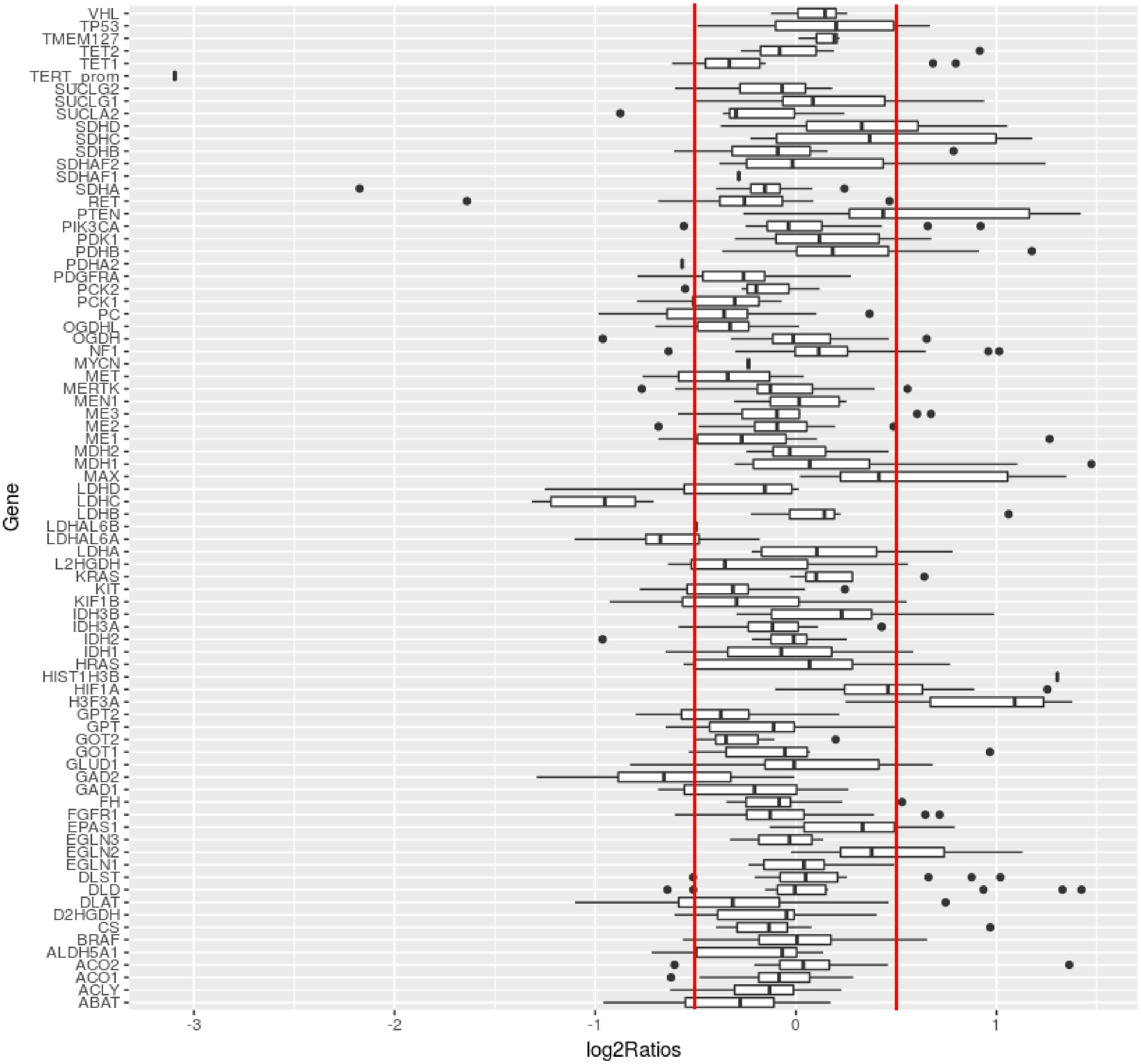

VHL
TP53
TMEM127
TET2
TET1
TERT_prom
SUCLG2
SUCLG1
SUCLA2
SDHD
SDHC
SDHB
SDHAF2
SDHAF1
SDHA
RET
PTEN
PIK3CA
PDK1
PDHB
PDHA2
PDGFRA
PCK2
PCK1
PC
OGDHL
OGDH
NF1
MYCN
MET
MERTK
MEN1
ME3
ME2
ME1
MDH2
MDH1
MAX
LDHD
LDHC
LDHB
LDHAL6B
LDHAL6A
LDHA
L2HGDH
KRAS
KIT
KIF1B
IDH3B
IDH3A
IDH2
IDH1
HRAS
HIST1H3B
HIF1A
H3F3A
GPT2
GPT
GOT2
GOT1
GLUD1
GAD2
GAD1
FH
FGFR1
EPAS1
EGLN3
EGLN2
EGLN1
DLST
DLD
DLAT
D2HGDH
CS
BRAF
ALDH5A1
ACO2
ACO1
ACLY
ABAT
Gene
-3
-2
-1
0
1
log2Ratios

ID70

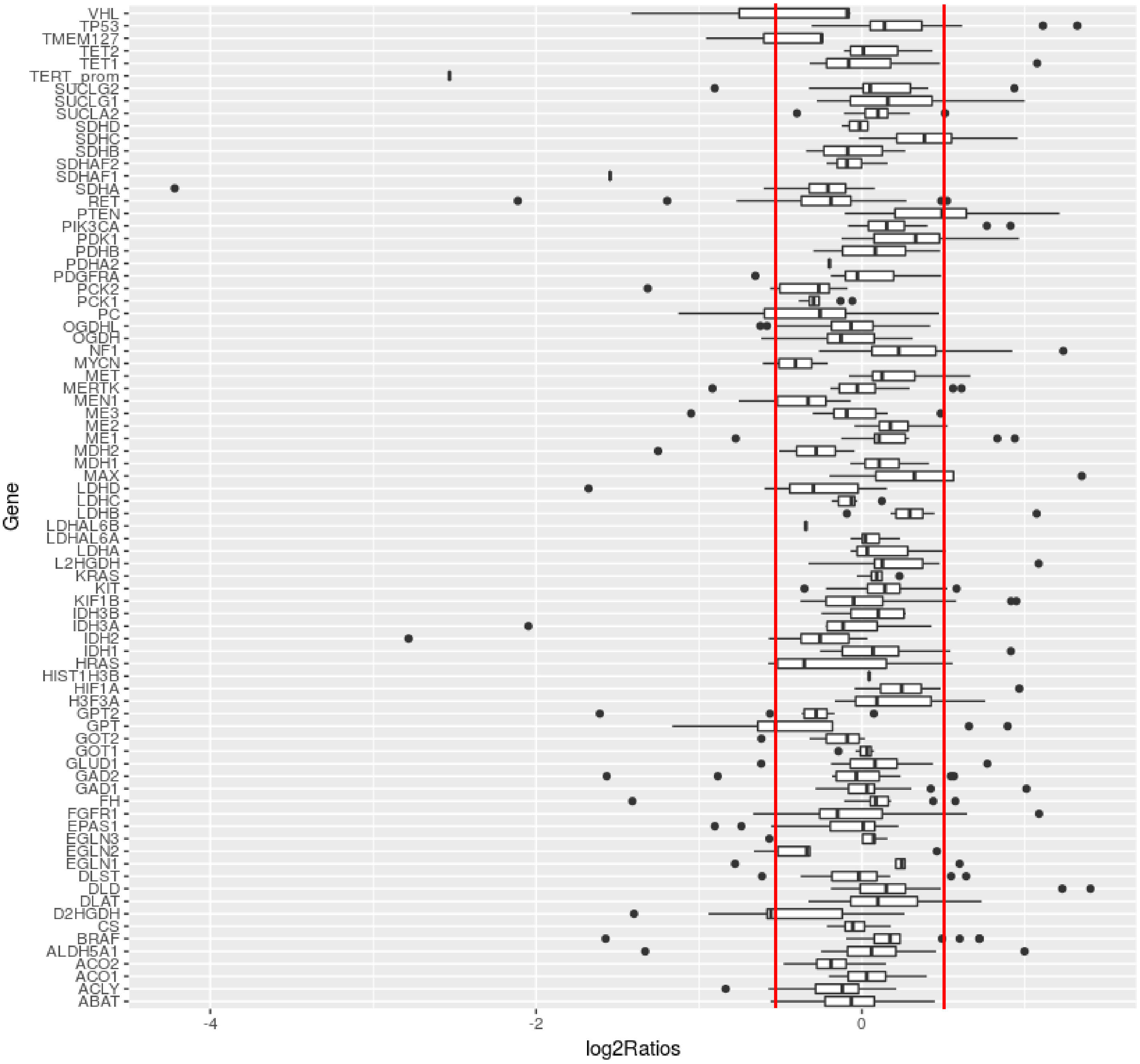

Gene
log2Ratios
-4
-2
0
VHL
TP53
TMEM127
TET2
TET1
TERT_prom
SUCLG2
SUCLG1
SUCLA2
SDHD
SDHC
SDHB
SDHAF2
SDHAF1
SDHA
RET
PTEN
PIK3CA
PDK1
PDHB
PDHA2
PDGFRA
PCK2
PCK1
PC
OGDHL
OGDH
NF1
MYCN
MET
MERTK
MEN1
ME3
ME2
ME1
MDH2
MDH1
MAX
LDHD
LDHC
LDHB
LDHAL6B
LDHAL6A
LDHA
L2HGDH
KRAS
KIT
KIF1B
IDH3B
IDH3A
IDH2
IDH1
HRAS
HIST1H3B
HIF1A
H3F3A
GPT2
GPT
GOT2
GOT1
GLUD1
GAD2
GAD1
FH
FGFR1
EPAS1
EGLN3
EGLN2
EGLN1
DLST
DLD
DLAT
D2HGDH
CS
BRAF
ALDH5A1
ACO2
ACO1
ACLY
ABAT

ID71

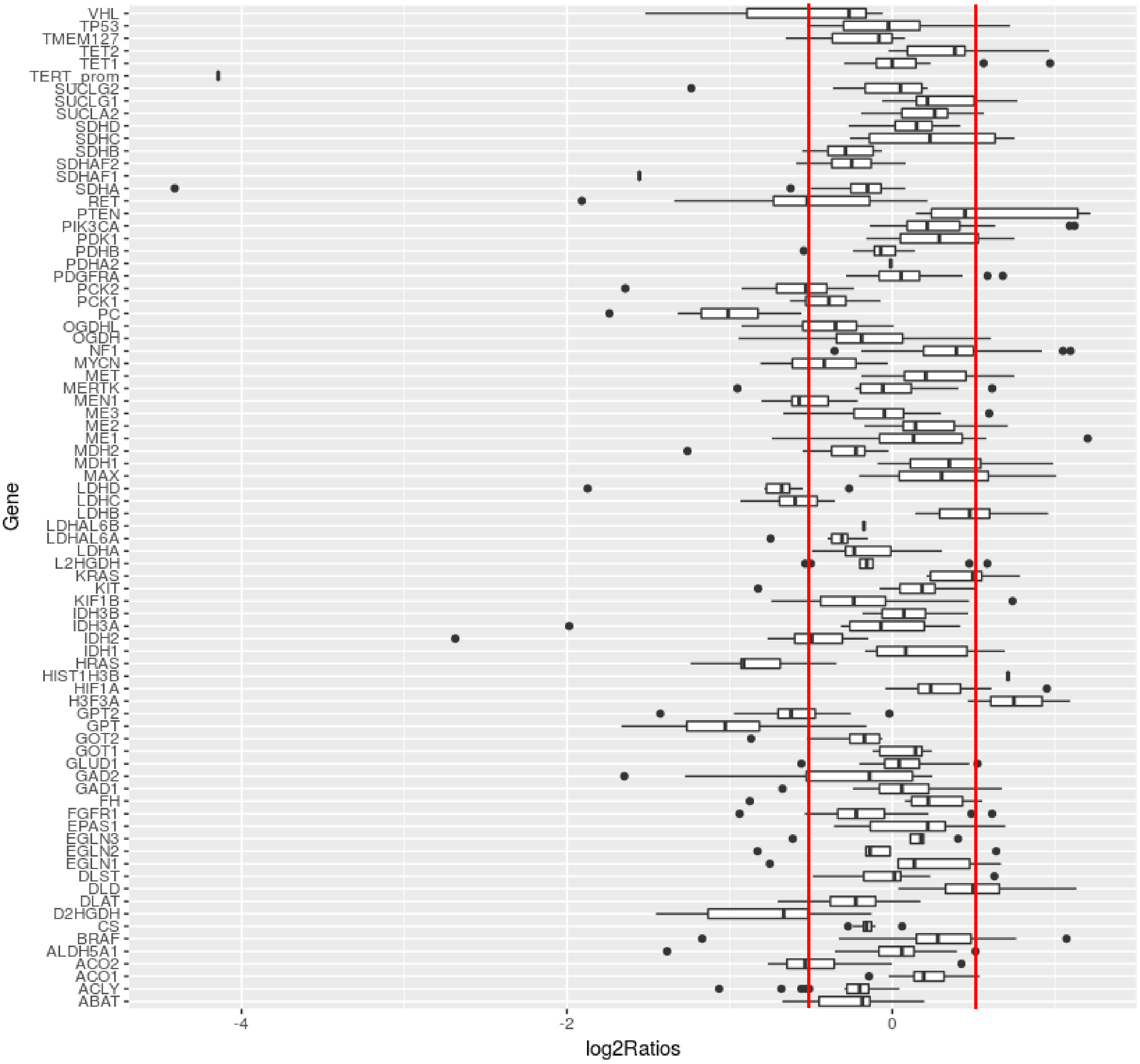
VHL
TP53
TMEM127
TET2
TET1
TERT_prom
SUCLG2
SUCLG1
SUCLA2
SDHD
SDHC
SDHB
SDHAF2
SDHAF1
SDHA
RET
PTEN
PIK3CA
PDK1
PDHB
PDHA2
PDGFRA
PCK2
PCK1
PC
OGDHL
OGDH
NF1
MYCN
MET
MERTK
MEN1
ME3
ME2
ME1
MDH2
MDH1
MAX
LDHD
LDHC
LDHB
LDHAL6B
LDHAL6A
LDHA
L2HGDH
KRAS
KIT
KIF1B
IDH3B
IDH3A
IDH2
IDH1
HRAS
HIST1H3B
HIF1A
H3F3A
GPT2
GPT
GOT2
GOT1
GLUD1
GAD2
GAD1
FH
FGFR1
EPAS1
EGLN3
EGLN2
EGLN1
DLST
DLD
DLAT
D2HGDH
CS
BRAF
ALDH5A1
ACO2
ACO1
ACLY
ABAT
Gene
-4
-2
0
log2Ratios

ID72

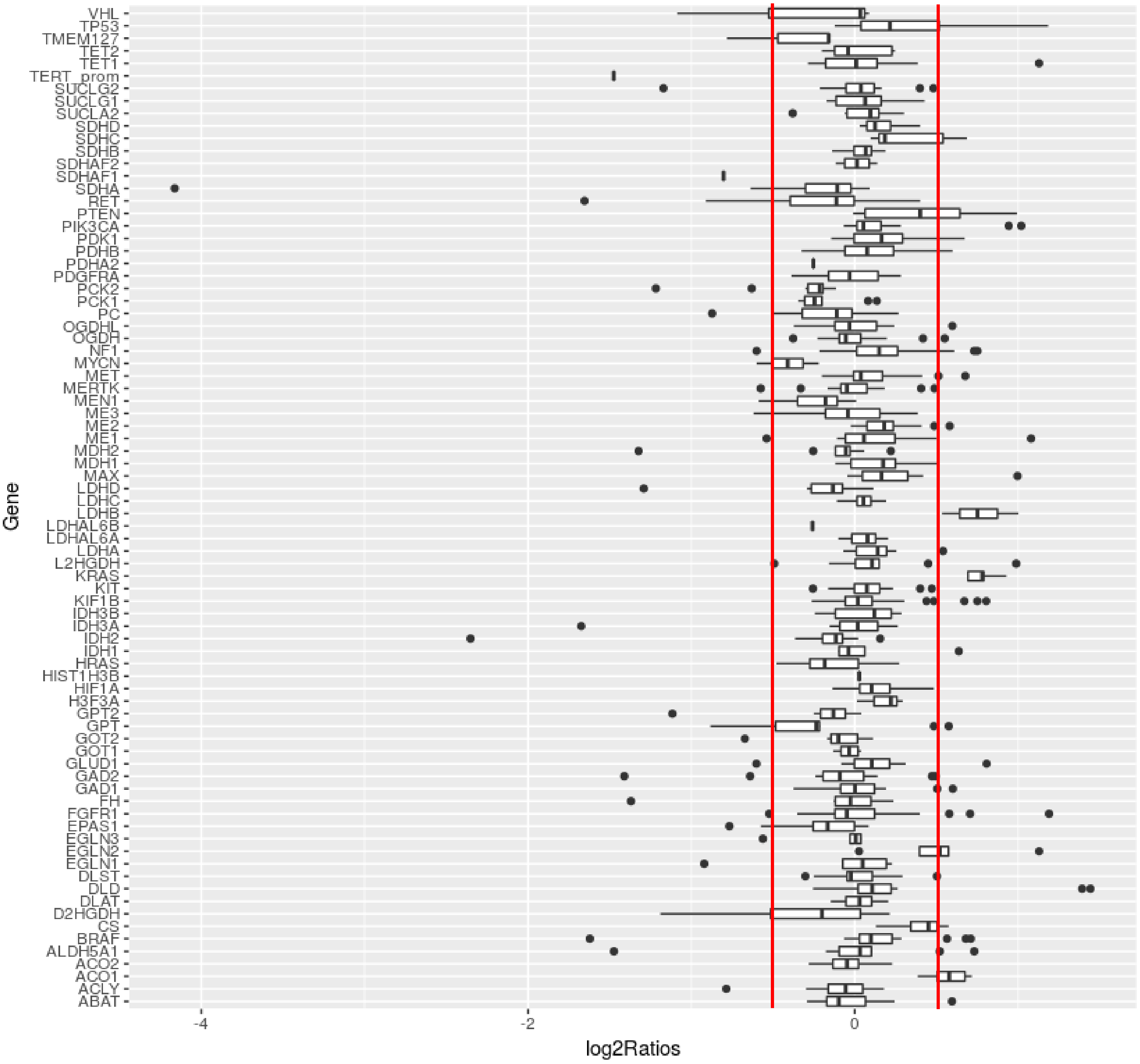

VHL
TP53
TMEM127
TET2
TET1
TERT_prom
SUCLG2
SUCLG1
SUCLA2
SDHD
SDHC
SDHB
SDHAF2
SDHAF1
SDHA
RET
PTEN
PIK3CA
PDK1
PDHB
PDHA2
PDGFRA
PCK2
PCK1
PC
OGDHL
OGDH
NF1
MYCN
MET
MERTK
MEN1
ME3
ME2
ME1
MDH2
MDH1
MAX
LDHD
LDHC
LDHB
LDHAL6B
LDHAL6A
LDHA
L2HGDH
KRAS
KIT
KIF1B
IDH3B
IDH3A
IDH2
IDH1
HRAS
HIST1H3B
HIF1A
H3F3A
GPT2
GPT
GOT2
GOT1
GLUD1
GAD2
GAD1
FH
FGFR1
EPAS1
EGLN3
EGLN2
EGLN1
DLST
DLD
DLAT
D2HGDH
CS
BRAF
ALDH5A1
ACO2
ACO1
ACLY
ABAT
Gene
-4
-2
0
log2Ratios

ID73

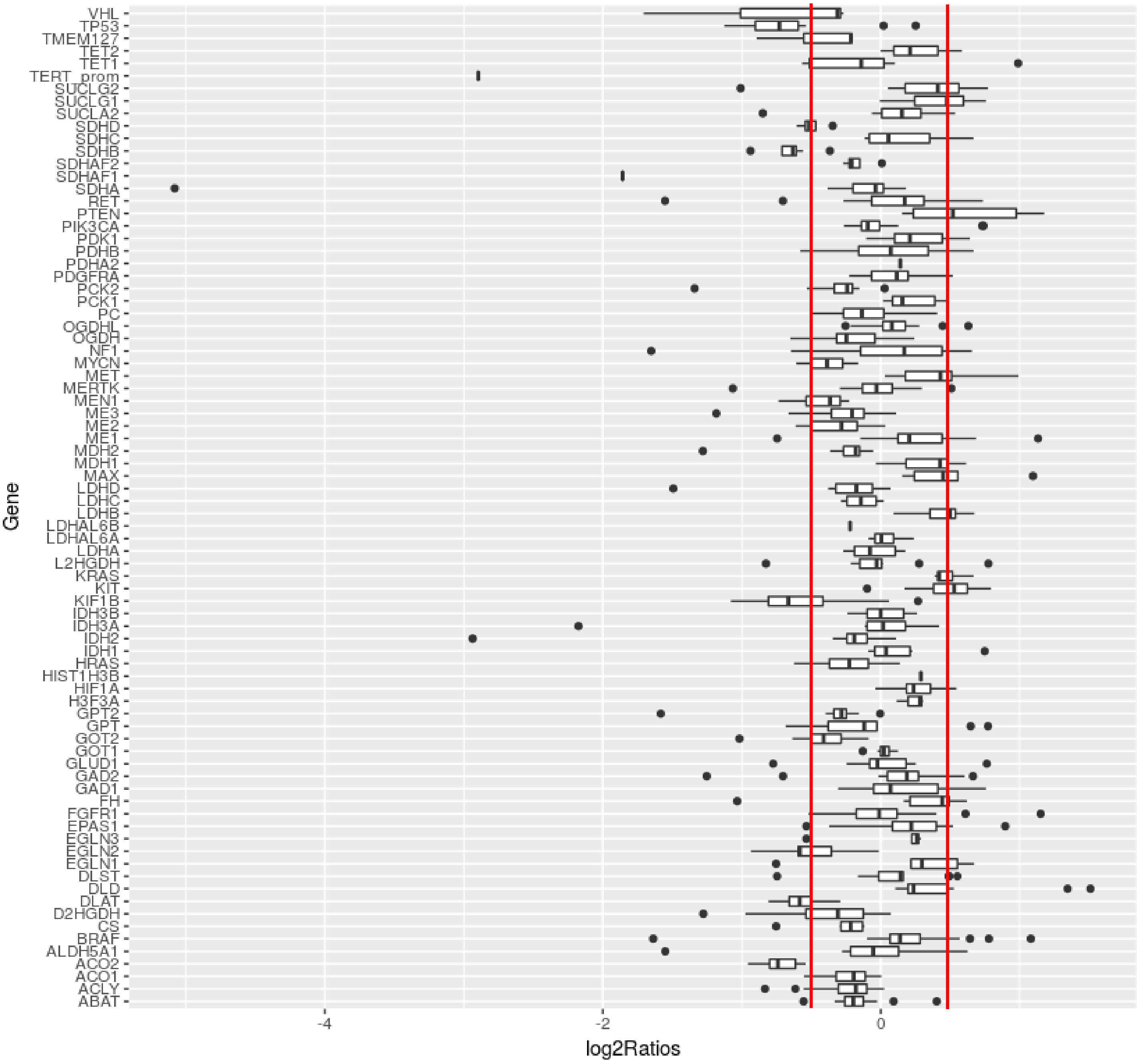
VHL
TP53
TMEM127
TET2
TET1
TERT_prom
SUCLG2
SUCLG1
SUCLA2
SDHD
SDHC
SDHB
SDHAF2
SDHAF1
SDHA
RET
PTEN
PIK3CA
PDK1
PDHB
PDHA2
PDGFRA
PCK2
PCK1
PC
OGDHL
OGDH
NF1
MYCN
MET
MERTK
MEN1
ME3
ME2
ME1
MDH2
MDH1
MAX
LDHD
LDHC
LDHB
LDHAL6B
LDHAL6A
LDHA
L2HGDH
KRAS
KIT
KIF1B
IDH3B
IDH3A
IDH2
IDH1
HRAS
HIST1H3B
HIF1A
H3F3A
GPT2
GPT
GOT2
GOT1
GLUD1
GAD2
GAD1
FH
FGFR1
EPAS1
EGLN3
EGLN2
EGLN1
DLST
DLD
DLAT
D2HGDH
CS
BRAF
ALDH5A1
ACO2
ACO1
ACLY
ABAT
Gene
-4
-2
0
log2Ratios

ID75

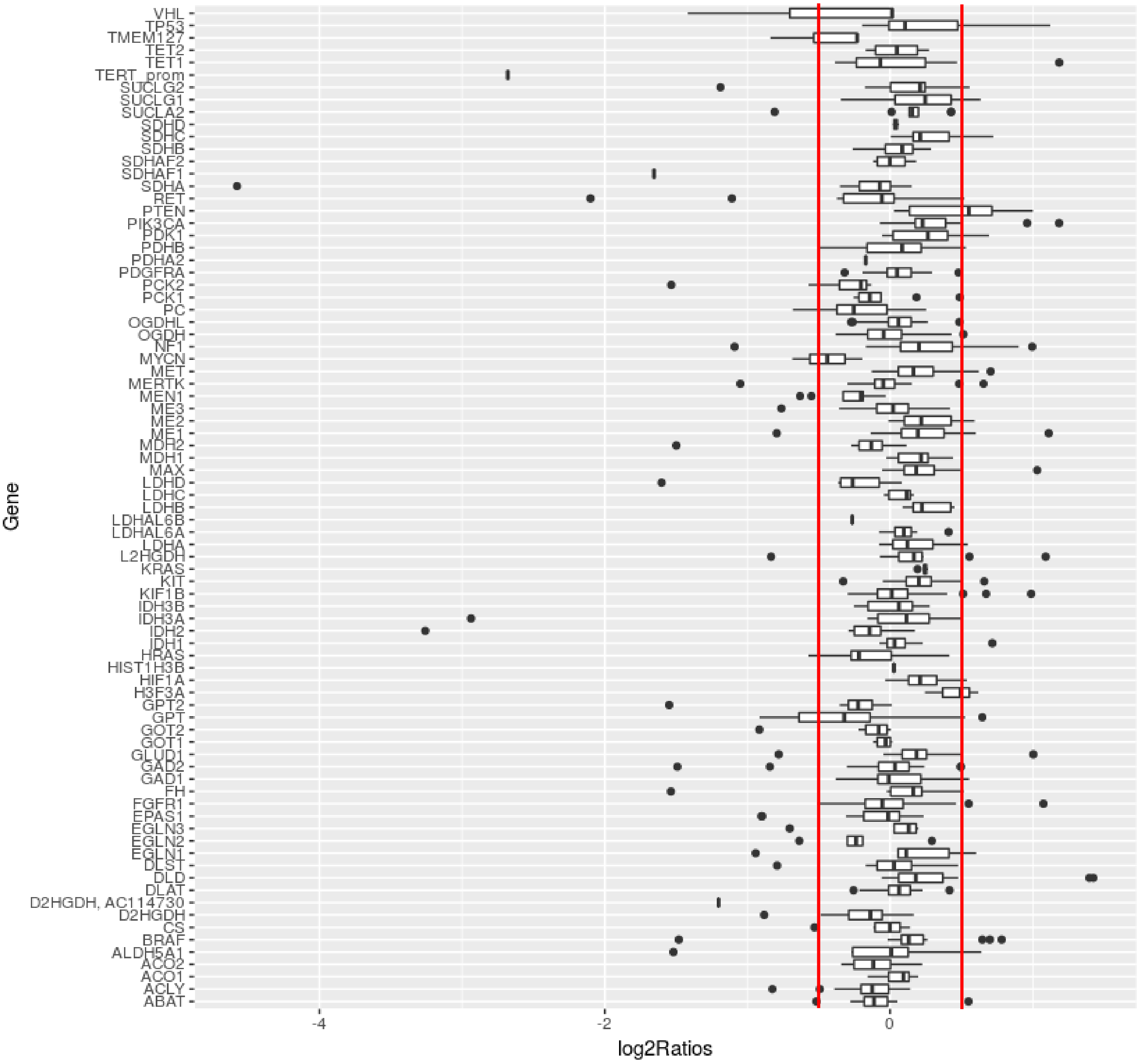

VHL
TP53
TMEM127
TET2
TET1
TERT_prom
SUCLG2
SUCLG1
SUCLA2
SDHD
SDHC
SDHB
SDHAF2
SDHAF1
SDHA
RET
PTEN
PIK3CA
PDK1
PDHB
PDHA2
PDGFRA
PCK2
PCK1
PC
OGDHL
OGDH
NF1
MYCN
MET
MERTK
MEN1
ME3
ME2
ME1
MDH2
MDH1
MAX
LDHD
LDHC
LDHB
LDHAL6B
LDHAL6A
LDHA
L2HGDH
KRAS
KIT
KIF1B
IDH3B
IDH3A
IDH2
IDH1
HRAS
HIST1H3B
HIF1A
H3F3A
GPT2
GPT
GOT2
GOT1
GLUD1
GAD2
GAD1
FH
FGFR1
EPAS1
EGLN3
EGLN2
EGLN1
DLST
DLD
DLAT
D2HGDH, AC114730
D2HGDH
CS
BRAF
ALDH5A1
ACO2
ACO1
ACLY
ABAT
Gene
-4
-2
0
log2Ratios

ID76

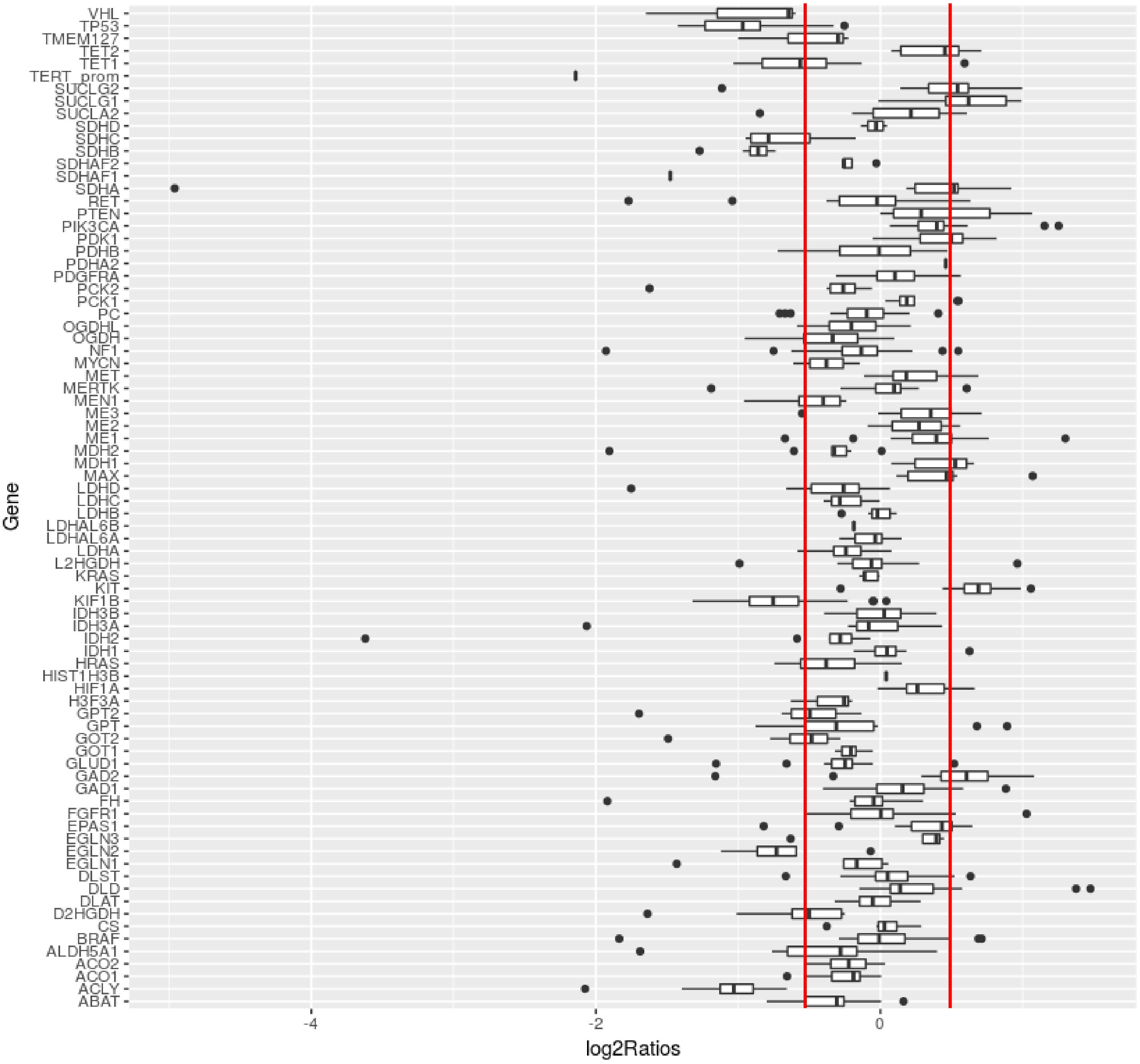

Gene
log2Ratios
-4
-2
0
VHL
TP53
TMEM127
TET2
TET1
TERT_prom
SUCLG2
SUCLG1
SUCLA2
SDHD
SDHC
SDHB
SDHAF2
SDHAF1
SDHA
RET
PTEN
PIK3CA
PDK1
PDHB
PDHA2
PDGFRA
PCK2
PCK1
PC
OGDHL
OGDH
NF1
MYCN
MET
MERTK
MEN1
ME3
ME2
ME1
MDH2
MDH1
MAX
LDHD
LDHC
LDHB
LDHAL6B
LDHAL6A
LDHA
L2HGDH
KRAS
KIT
KIF1B
IDH3B
IDH3A
IDH2
IDH1
HRAS
HIST1H3B
HIF1A
H3F3A
GPT2
GPT
GOT2
GOT1
GLUD1
GAD2
GAD1
FH
FGFR1
EPAS1
EGLN3
EGLN2
EGLN1
DLST
DLD
DLAT
D2HGDH
CS
BRAF
ALDH5A1
ACO2
ACO1
ACLY
ABAT

ID78

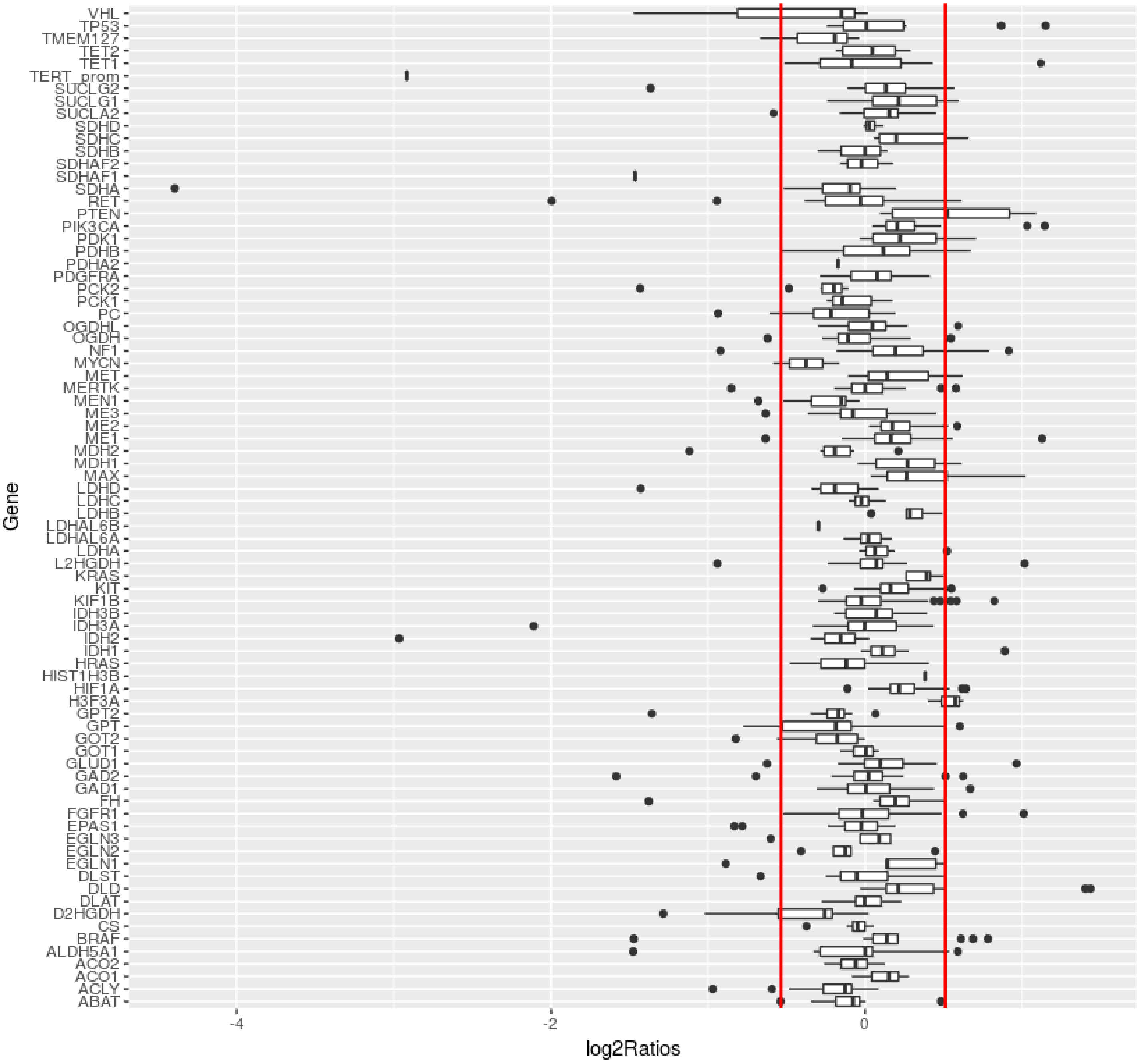

VHL
TP53
TMEM127
TET2
TET1
TERT_prom
SUCLG2
SUCLG1
SUCLA2
SDHD
SDHC
SDHB
SDHAF2
SDHAF1
SDHA
RET
PTEN
PIK3CA
PDK1
PDHB
PDHA2
PDGFRA
PCK2
PCK1
PC
OGDHL
OGDH
NF1
MYCN
MET
MERTK
MEN1
ME3
ME2
ME1
MDH2
MDH1
MAX
LDHD
LDHC
LDHB
LDHAL6B
LDHAL6A
LDHA
L2HGDH
KRAS
KIT
KIF1B
IDH3B
IDH3A
IDH2
IDH1
HRAS
HIST1H3B
HIF1A
H3F3A
GPT2
GPT
GOT2
GOT1
GLUD1
GAD2
GAD1
FH
FGFR1
EPAS1
EGLN3
EGLN2
EGLN1
DLST
DLD
DLAT
D2HGDH
CS
BRAF
ALDH5A1
ACO2
ACO1
ACLY
ABAT
Gene
-4
-2
0
log2Ratios

ID79

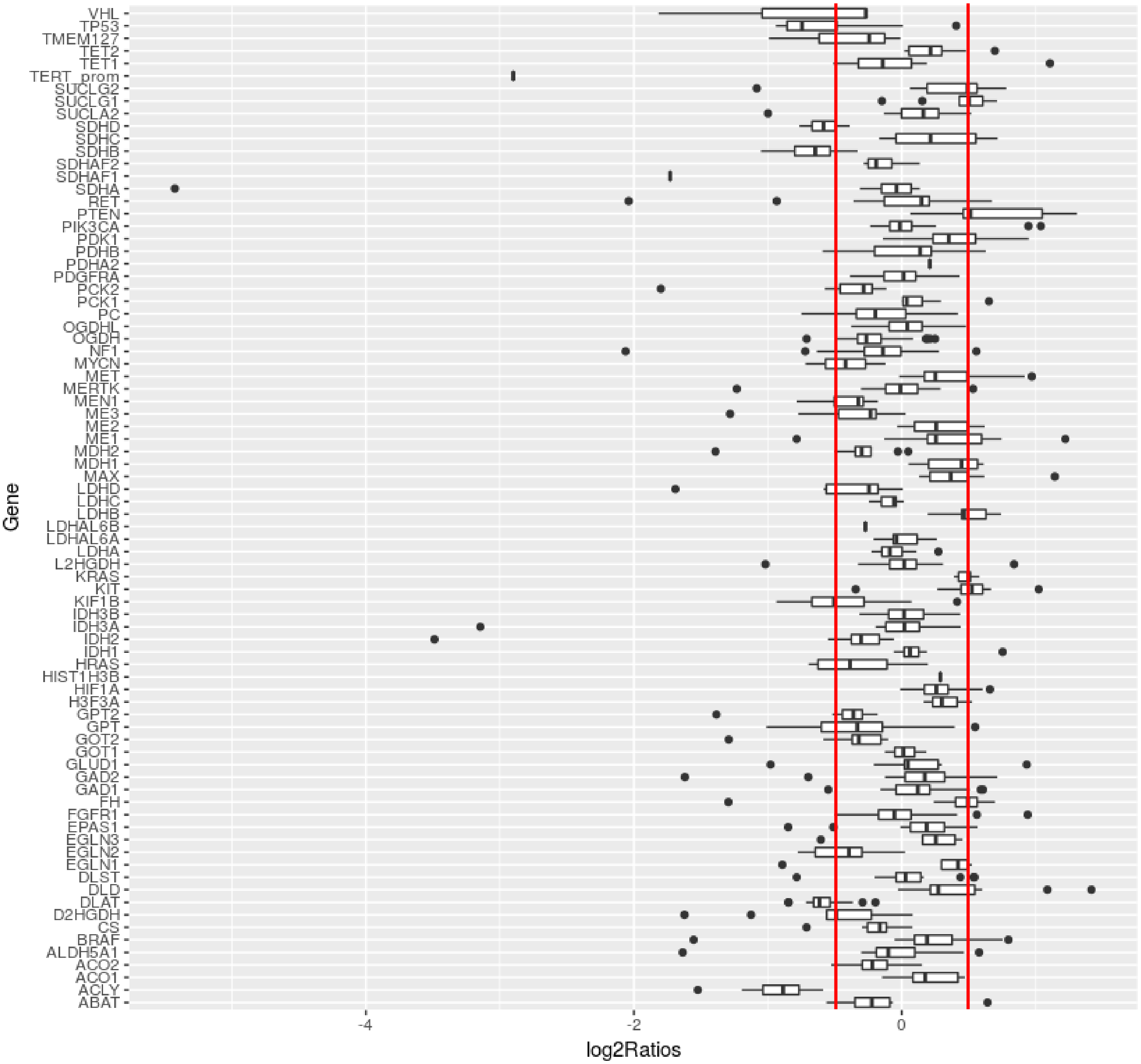

Gene
log2Ratios
-4
-2
0
VHL
TP53
TMEM127
TET2
TET1
TERT_prom
SUCLG2
SUCLG1
SUCLA2
SDHD
SDHC
SDHB
SDHAF2
SDHAF1
SDHA
RET
PTEN
PIK3CA
PDK1
PDHB
PDHA2
PDGFRA
PCK2
PCK1
PC
OGDHL
OGDH
NF1
MYCN
MET
MERTK
MEN1
ME3
ME2
ME1
MDH2
MDH1
MAX
LDHD
LDHC
LDHB
LDHAL6B
LDHAL6A
LDHA
L2HGDH
KRAS
KIT
KIF1B
IDH3B
IDH3A
IDH2
IDH1
HRAS
HIST1H3B
HIF1A
H3F3A
GPT2
GPT
GOT2
GOT1
GLUD1
GAD2
GAD1
FH
FGFR1
EPAS1
EGLN3
EGLN2
EGLN1
DLST
DLD
DLAT
D2HGDH
CS
BRAF
ALDH5A1
ACO2
ACO1
ACLY
ABAT

ID80

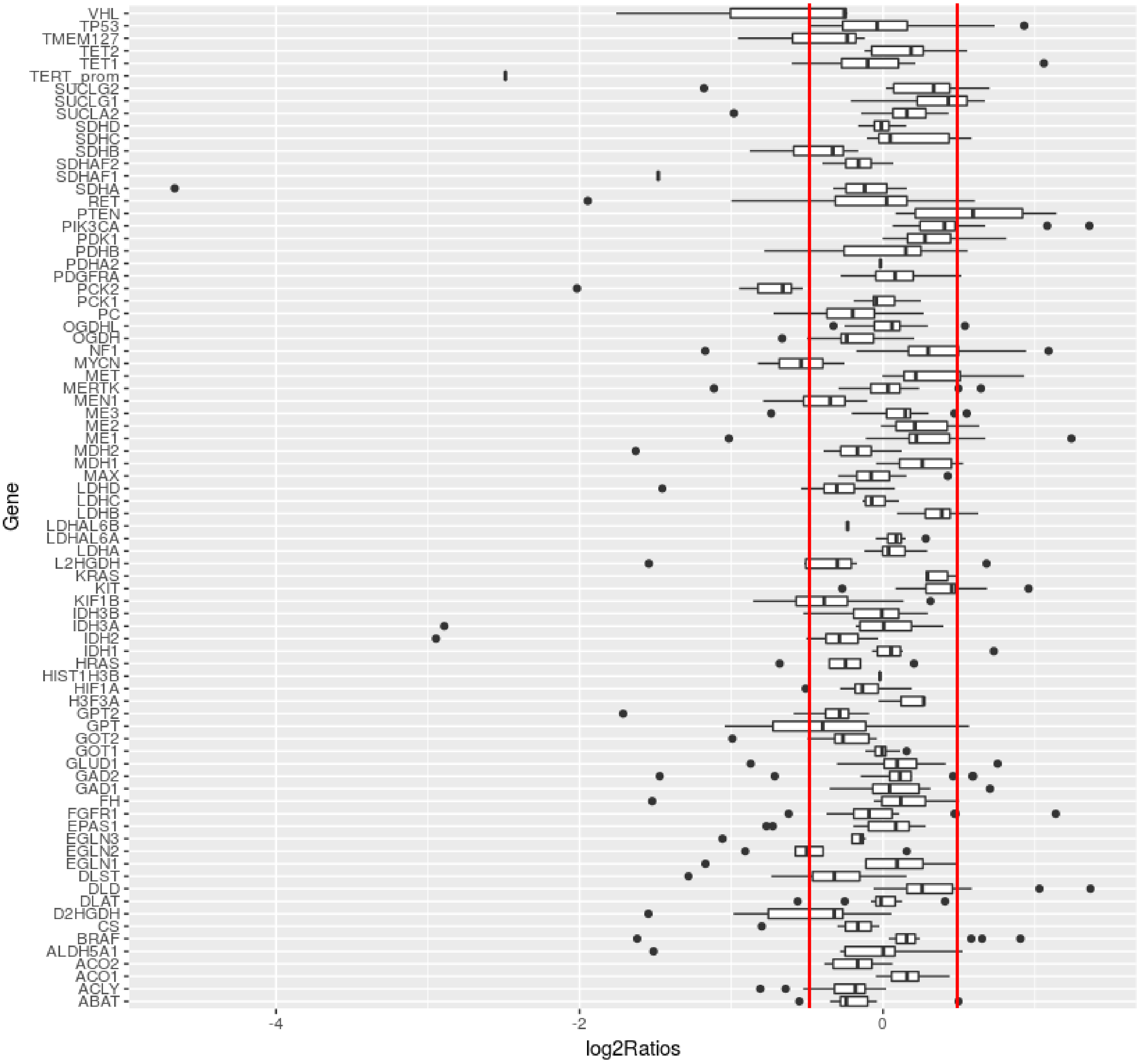

VHL
TP53
TMEM127
TET2
TET1
TERT_prom
SUCLG2
SUCLG1
SUCLA2
SDHD
SDHC
SDHB
SDHAF2
SDHAF1
SDHA
RET
PTEN
PIK3CA
PDK1
PDHB
PDHA2
PDGFRA
PCK2
PCK1
PC
OGDHL
OGDH
NF1
MYCN
MET
MERTK
MEN1
ME3
ME2
ME1
MDH2
MDH1
MAX
LDHD
LDHC
LDHB
LDHAL6B
LDHAL6A
LDHA
L2HGDH
KRAS
KIT
KIF1B
IDH3B
IDH3A
IDH2
IDH1
HRAS
HIST1H3B
HIF1A
H3F3A
GPT2
GPT
GOT2
GOT1
GLUD1
GAD2
GAD1
FH
FGFR1
EPAS1
EGLN3
EGLN2
EGLN1
DLST
DLD
DLAT
D2HGDH
CS
BRAF
ALDH5A1
ACO2
ACO1
ACLY
ABAT
Gene
-4
-2
0
log2Ratios

ID82

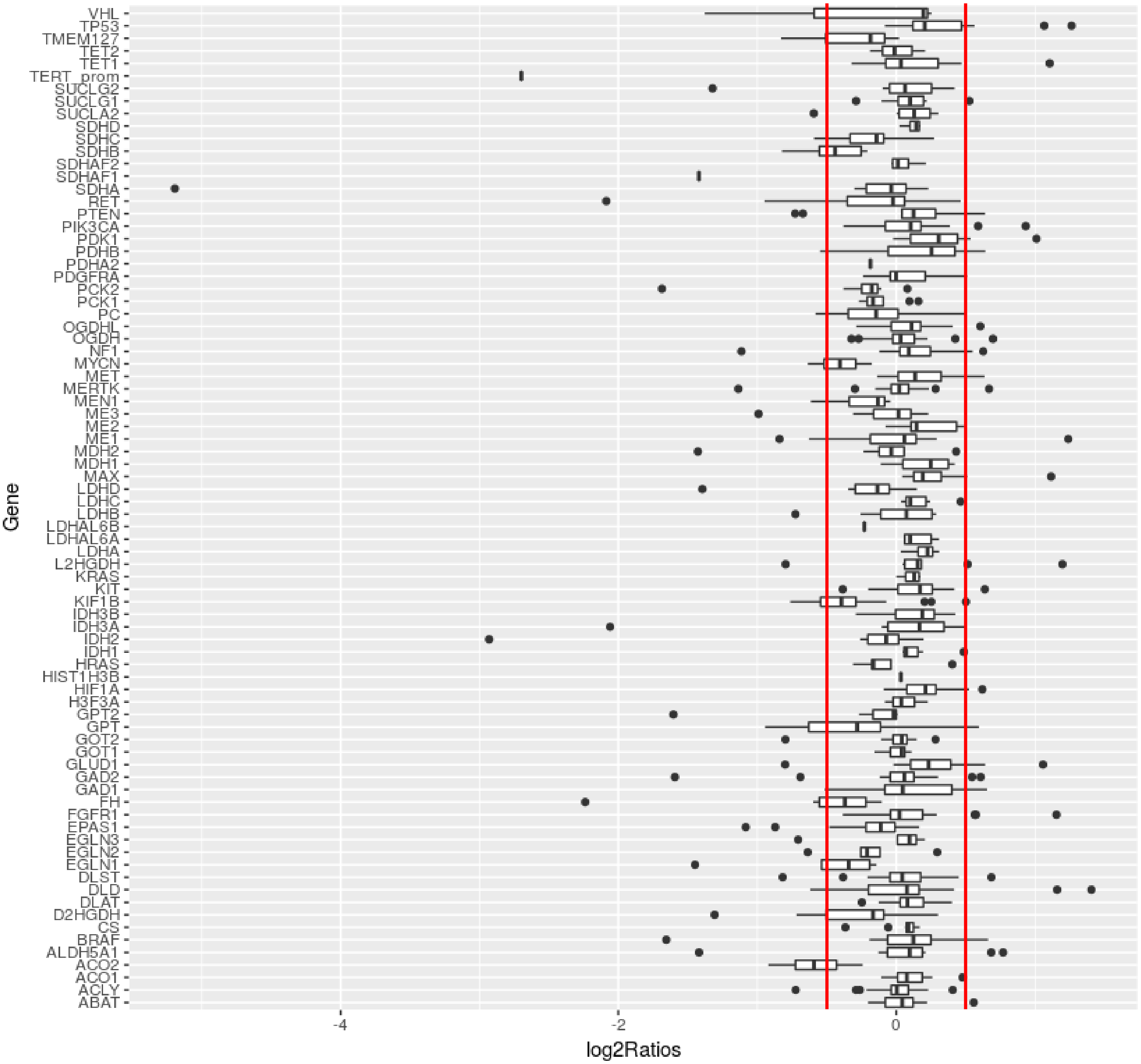

Gene
log2Ratios
-4
-2
0
VHL
TP53
TMEM127
TET2
TET1
TERT_prom
SUCLG2
SUCLG1
SUCLA2
SDHD
SDHC
SDHB
SDHAF2
SDHAF1
SDHA
RET
PTEN
PIK3CA
PDK1
PDHB
PDHA2
PDGFRA
PCK2
PCK1
PC
OGDHL
OGDH
NF1
MYCN
MET
MERTK
MEN1
ME3
ME2
ME1
MDH2
MDH1
MAX
LDHD
LDHC
LDHB
LDHAL6B
LDHAL6A
LDHA
L2HGDH
KRAS
KIT
KIF1B
IDH3B
IDH3A
IDH2
IDH1
HRAS
HIST1H3B
HIF1A
H3F3A
GPT2
GPT
GOT2
GOT1
GLUD1
GAD2
GAD1
FH
FGFR1
EPAS1
EGLN3
EGLN2
EGLN1
DLST
DLD
DLAT
D2HGDH
CS
BRAF
ALDH5A1
ACO2
ACO1
ACLY
ABAT

ID88

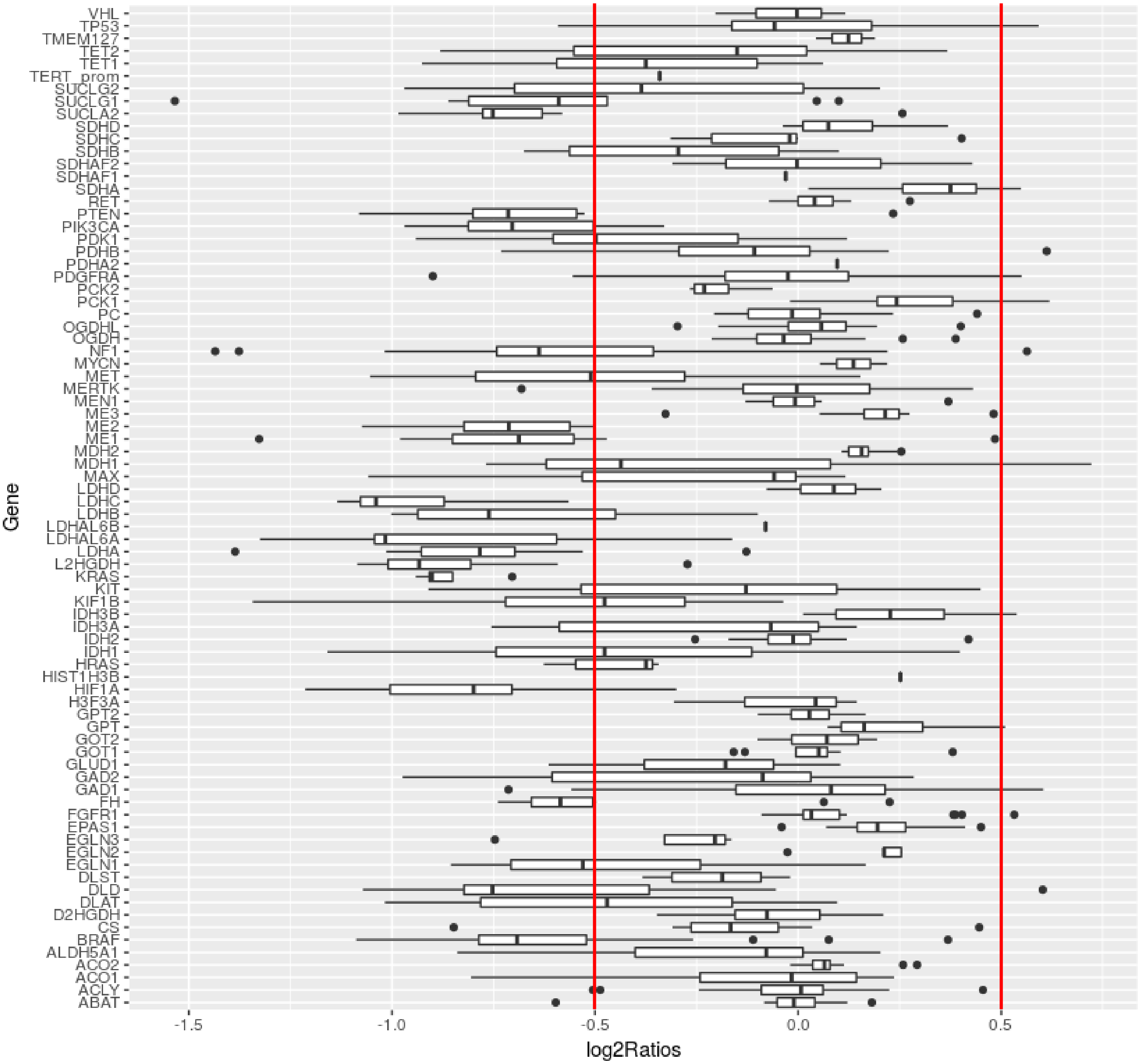
VHL
TP53
TMEM127
TET2
TET1
TERT_prom
SUCLG2
SUCLG1
SUCLA2
SDHD
SDHC
SDHB
SDHAF2
SDHAF1
SDHA
RET
PTEN
PIK3CA
PDK1
PDHB
PDHA2
PDGFRA
PCK2
PCK1
PC
OGDHL
OGDH
NF1
MYCN
MET
MERTK
MEN1
ME3
ME2
ME1
MDH2
MDH1
MAX
LDHD
LDHC
LDHB
LDHAL6B
LDHAL6A
LDHA
L2HGDH
KRAS
KIT
KIF1B
IDH3B
IDH3A
IDH2
IDH1
HRAS
HIST1H3B
HIF1A
H3F3A
GPT2
GPT
GOT2
GOT1
GLUD1
GAD2
GAD1
FH
FGFR1
EPAS1
EGLN3
EGLN2
EGLN1
DLST
DLD
DLAT
D2HGDH
CS
BRAF
ALDH5A1
ACO2
ACO1
ACLY
ABAT
Gene
-1.5
-1.0
-0.5
0.0
0.5
log2Ratios

ID89

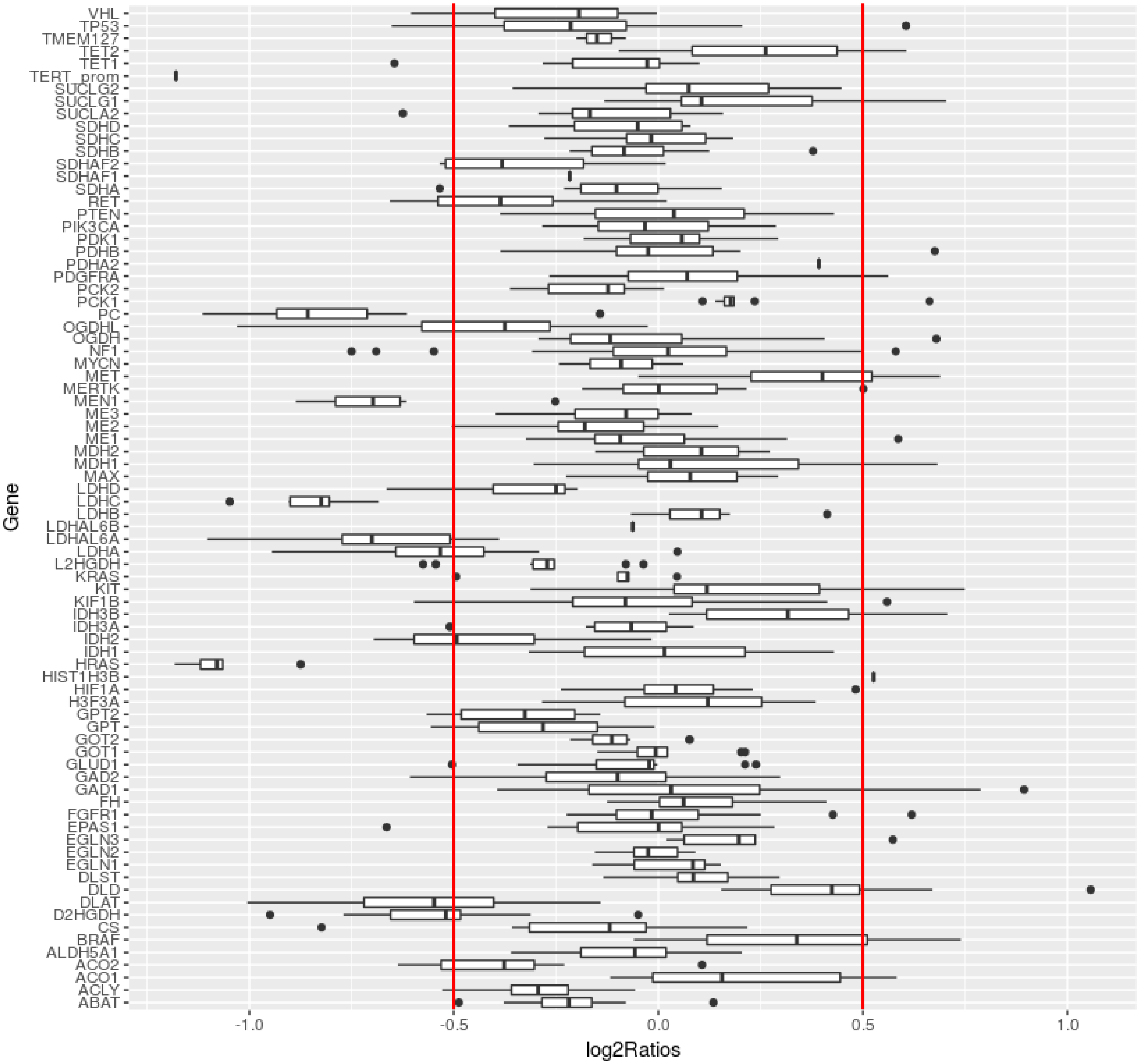

VHL
TP53
TMEM127
TET2
TET1
TERT_prom
SUCLG2
SUCLG1
SUCLA2
SDHD
SDHC
SDHB
SDHAF2
SDHAF1
SDHA
RET
PTEN
PIK3CA
PDK1
PDHB
PDHA2
PDGFRA
PCK2
PCK1
PC
OGDHL
OGDH
NF1
MYCN
MET
MERTK
MEN1
ME3
ME2
ME1
MDH2
MDH1
MAX
LDHD
LDHC
LDHB
LDHAL6B
LDHAL6A
LDHA
L2HGDH
KRAS
KIT
KIF1B
IDH3B
IDH3A
IDH2
IDH1
HRAS
HIST1H3B
HIF1A
H3F3A
GPT2
GPT
GOT2
GOT1
GLUD1
GAD2
GAD1
FH
FGFR1
EPAS1
EGLN3
EGLN2
EGLN1
DLST
DLD
DLAT
D2HGDH
CS
BRAF
ALDH5A1
ACO2
ACO1
ACLY
ABAT
Gene
-1.0
-0.5
0.0
0.5
1.0
log2Ratios

ID90

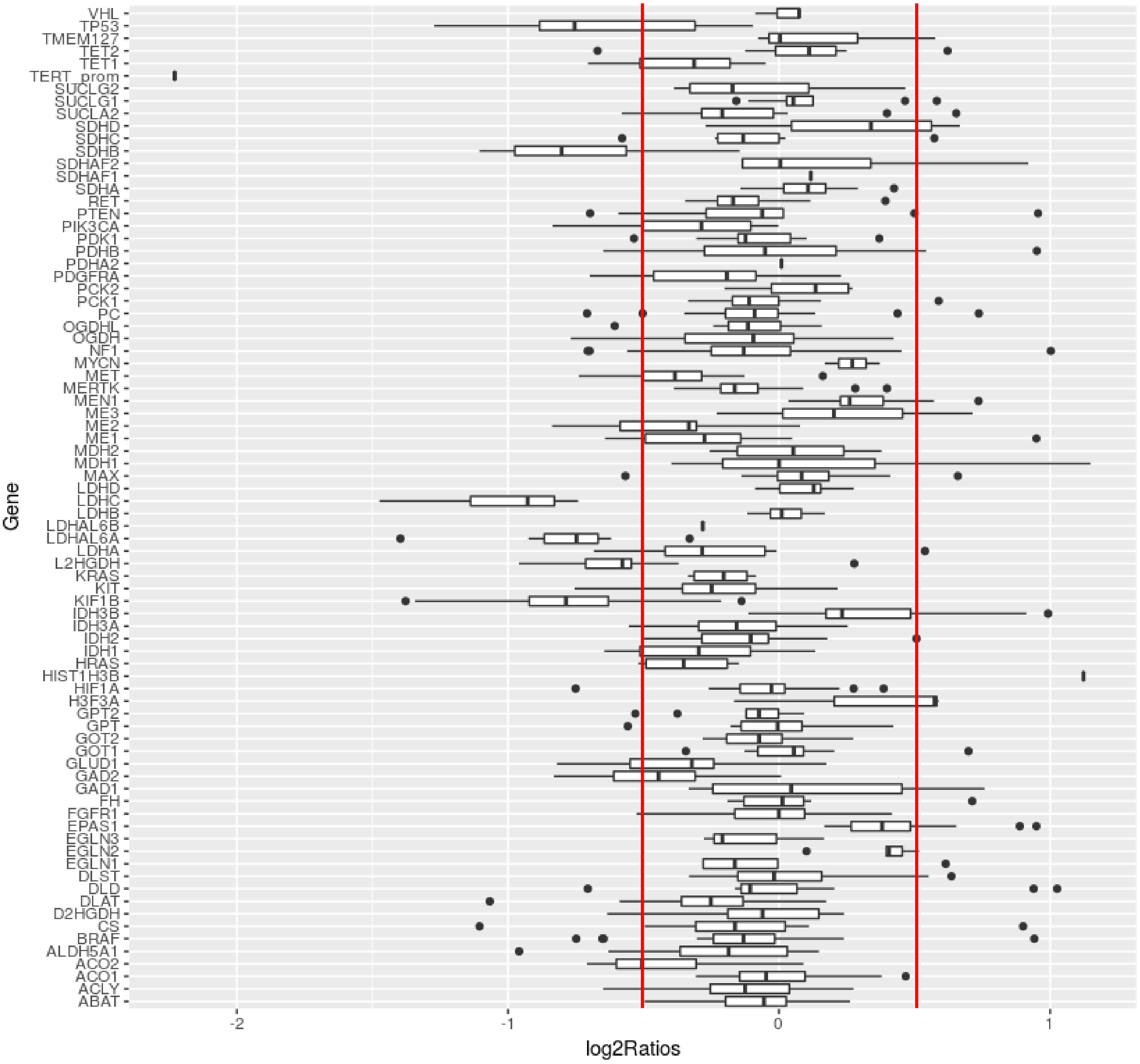

VHL
TP53
TMEM127
TET2
TET1
TERT_prom
SUCLG2
SUCLG1
SUCLA2
SDHD
SDHC
SDHB
SDHAF2
SDHAF1
SDHA
RET
PTEN
PIK3CA
PDK1
PDHB
PDHA2
PDGFRA
PCK2
PCK1
PC
OGDHL
OGDH
NF1
MYCN
MET
MERTK
MEN1
ME3
ME2
ME1
MDH2
MDH1
MAX
LDHD
LDHC
LDHB
LDHAL6B
LDHAL6A
LDHA
L2HGDH
KRAS
KIT
KIF1B
IDH3B
IDH3A
IDH2
IDH1
HRAS
HIST1H3B
HIF1A
H3F3A
GPT2
GPT
GOT2
GOT1
GLUD1
GAD2
GAD1
FH
FGFR1
EPAS1
EGLN3
EGLN2
EGLN1
DLST
DLD
DLAT
D2HGDH
CS
BRAF
ALDH5A1
ACO2
ACO1
ACLY
ABAT
Gene
-2
-1
0
1
log2Ratios

ID91

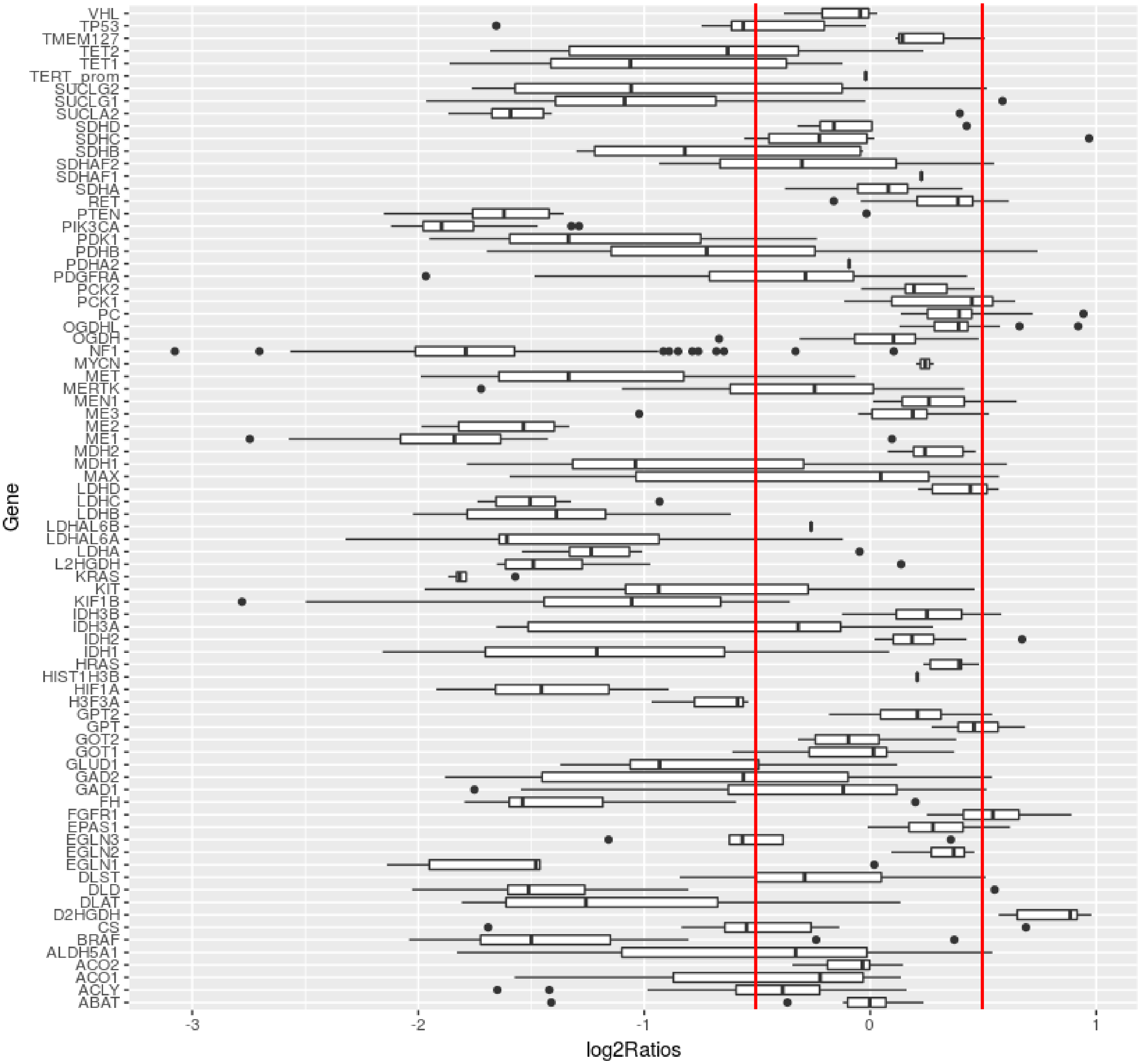

Gene
log2Ratios
VHL
TP53
TMEM127
TET2
TET1
TERT_prom
SUCLG2
SUCLG1
SUCLA2
SDHD
SDHC
SDHB
SDHAF2
SDHAF1
SDHA
RET
PTEN
PIK3CA
PDK1
PDHB
PDHA2
PDGFRA
PCK2
PCK1
PC
OGDHL
OGDH
NF1
MYCN
MET
MERTK
MEN1
ME3
ME2
ME1
MDH2
MDH1
MAX
LDHD
LDHC
LDHB
LDHAL6B
LDHAL6A
LDHA
L2HGDH
KRAS
KIT
KIF1B
IDH3B
IDH3A
IDH2
IDH1
HRAS
HIST1H3B
HIF1A
H3F3A
GPT2
GPT
GOT2
GOT1
GLUD1
GAD2
GAD1
FH
FGFR1
EPAS1
EGLN3
EGLN2
EGLN1
DLST
DLD
DLAT
D2HGDH
CS
BRAF
ALDH5A1
ACO2
ACO1
ACLY
ABAT
-3
-2
-1
0
1

ID92

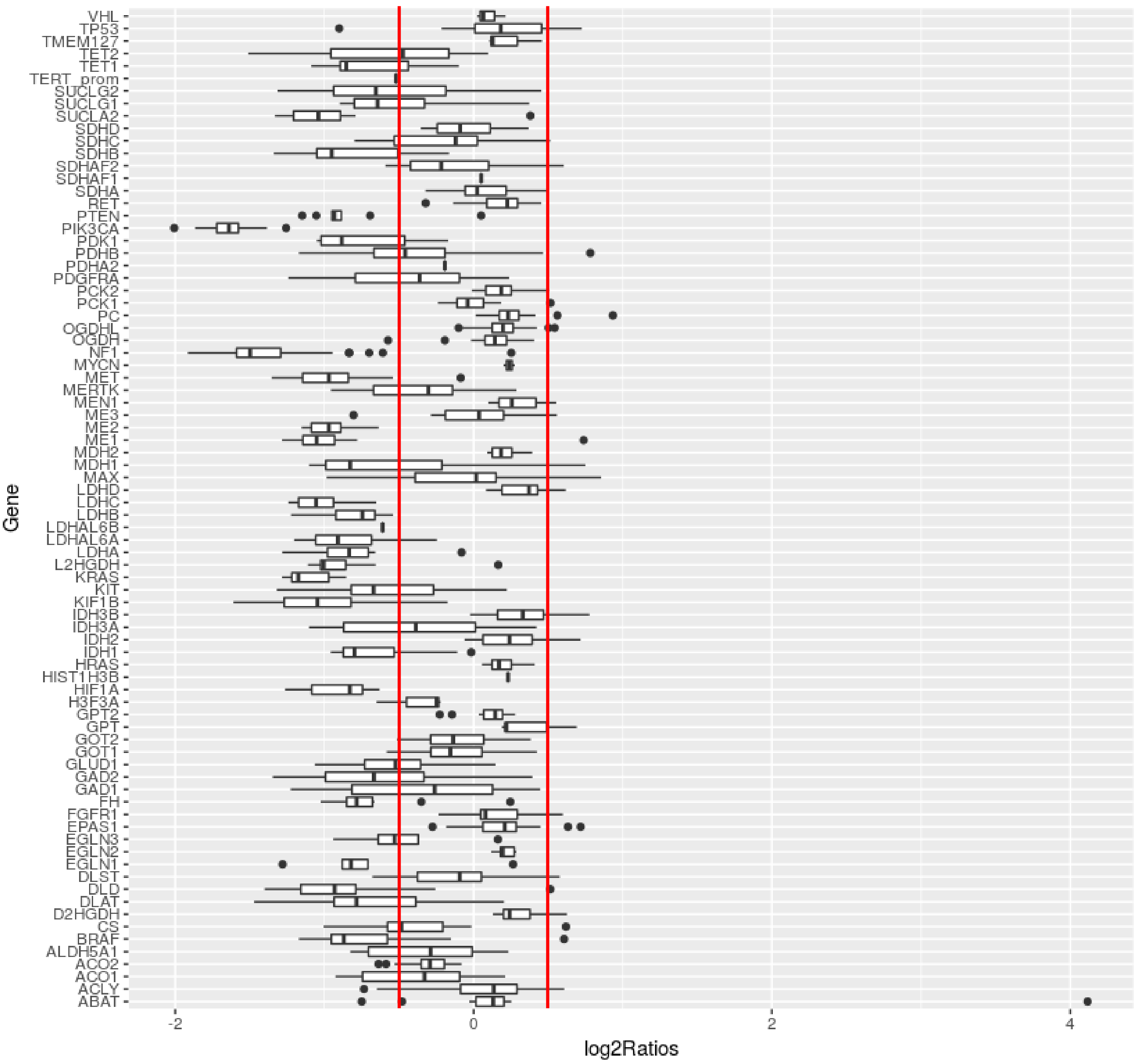
VHL
TP53
TMEM127
TET2
TET1
TERT_prom
SUCLG2
SUCLG1
SUCLA2
SDHD
SDHC
SDHB
SDHAF2
SDHAF1
SDHA
RET
PTEN
PIK3CA
PDK1
PDHB
PDHA2
PDGFRA
PCK2
PCK1
PC
OGDHL
OGDH
NF1
MYCN
MET
MERTK
MEN1
ME3
ME2
ME1
MDH2
MDH1
MAX
LDHD
LDHC
LDHB
LDHAL6B
LDHAL6A
LDHA
L2HGDH
KRAS
KIT
KIF1B
IDH3B
IDH3A
IDH2
IDH1
HRAS
HIST1H3B
HIF1A
H3F3A
GPT2
GPT
GOT2
GOT1
GLUD1
GAD2
GAD1
FH
FGFR1
EPAS1
EGLN3
EGLN2
EGLN1
DLST
DLD
DLAT
D2HGDH
CS
BRAF
ALDH5A1
ACO2
ACO1
ACLY
ABAT
Gene
-2
0
2
4
log2Ratios

delNF1 control

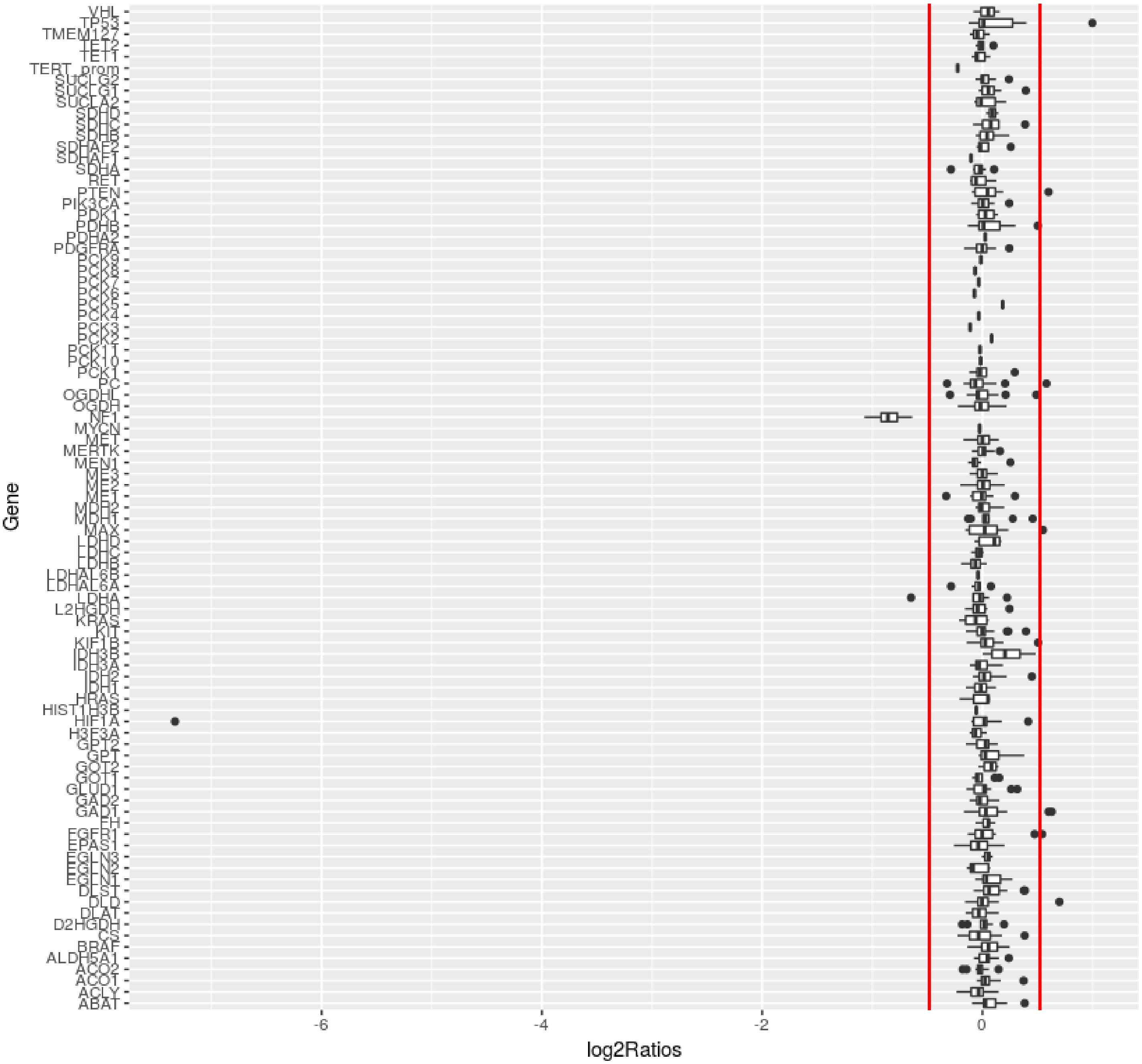

Control 1

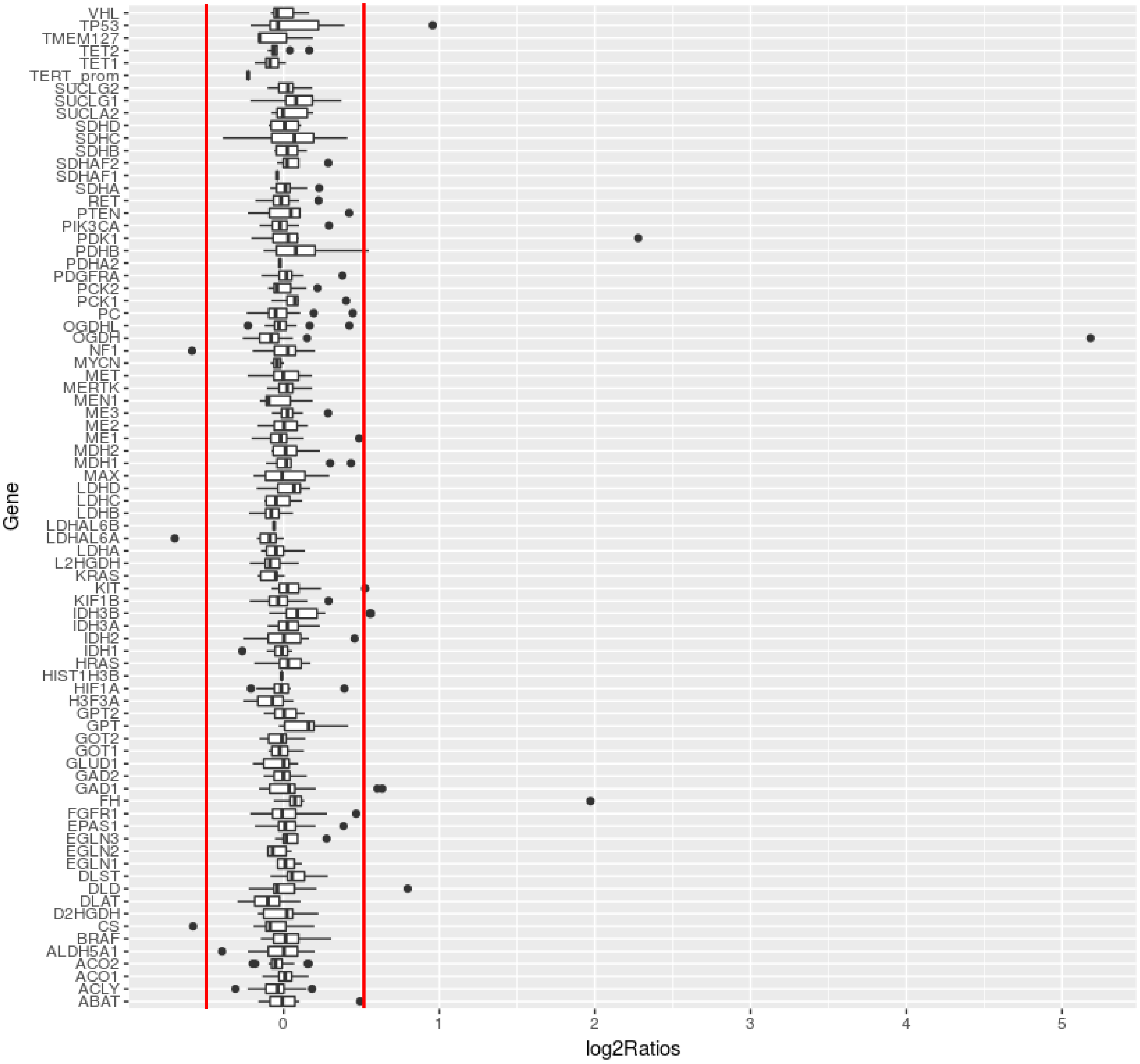

VHL
TP53
TMEM127
TET2
TET1
TERT_prom
SUCLG2
SUCLG1
SUCLA2
SDHD
SDHC
SDHB
SDHAF2
SDHAF1
SDHA
RET
PTEN
PIK3CA
PDK1
PDHB
PDHA2
PDGFRA
PCK2
PCK1
PC
OGDHL
OGDH
NF1
MYCN
MET
MERTK
MEN1
ME3
ME2
ME1
MDH2
MDH1
MAX
LDHD
LDHC
LDHB
LDHAL6B
LDHAL6A
LDHA
L2HGDH
KRAS
KIT
KIF1B
IDH3B
IDH3A
IDH2
IDH1
HRAS
HIST1H3B
HIF1A
H3F3A
GPT2
GPT
GOT2
GOT1
GLUD1
GAD2
GAD1
FH
FGFR1
EPAS1
EGLN3
EGLN2
EGLN1
DLST
DLD
DLAT
D2HGDH
CS
BRAF
ALDH5A1
ACO2
ACO1
ACLY
ABAT
Gene
0
1
2
3
4
5
log2Ratios

Control 2

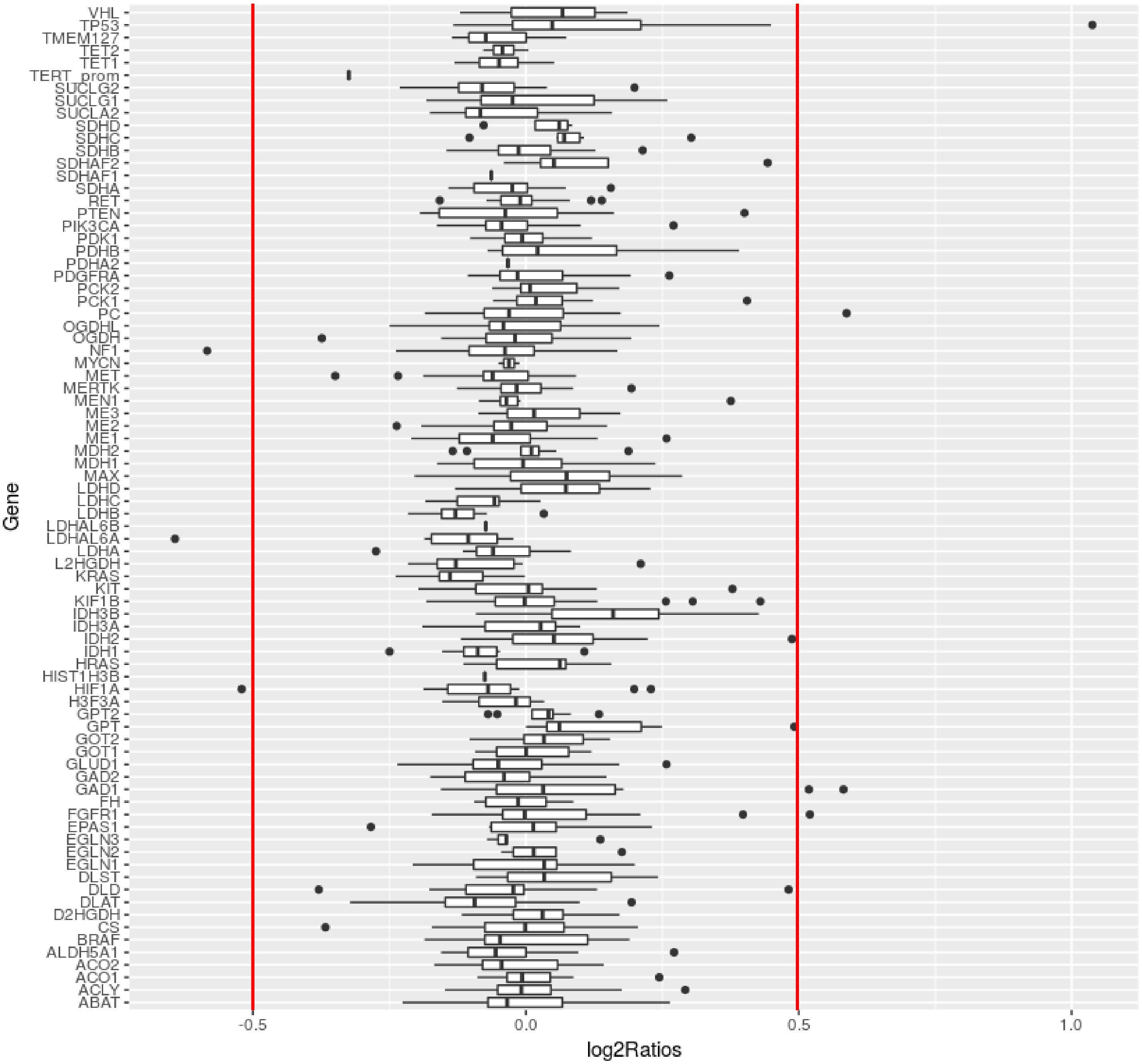

VHL
TP53
TMEM127
TET2
TET1
TERT_prom
SUCLG2
SUCLG1
SUCLA2
SDHD
SDHC
SDHB
SDHAF2
SDHAF1
SDHA
RET
PTEN
PIK3CA
PDK1
PDHB
PDHA2
PDGFRA
PCK2
PCK1
PC
OGDHL
OGDH
NF1
MYCN
MET
MERTK
MEN1
ME3
ME2
ME1
MDH2
MDH1
MAX
LDHD
LDHC
LDHB
LDHAL6B
LDHAL6A
LDHA
L2HGDH
KRAS
KIT
KIF1B
IDH3B
IDH3A
IDH2
IDH1
HRAS
HIST1H3B
HIF1A
H3F3A
GPT2
GPT
GOT2
GOT1
GLUD1
GAD2
GAD1
FH
FGFR1
EPAS1
EGLN3
EGLN2
EGLN1
DLST
DLD
DLAT
D2HGDH
CS
BRAF
ALDH5A1
ACO2
ACO1
ACLY
ABAT
Gene
-0.5
0.0
0.5
1.0
log2Ratios

Control 3

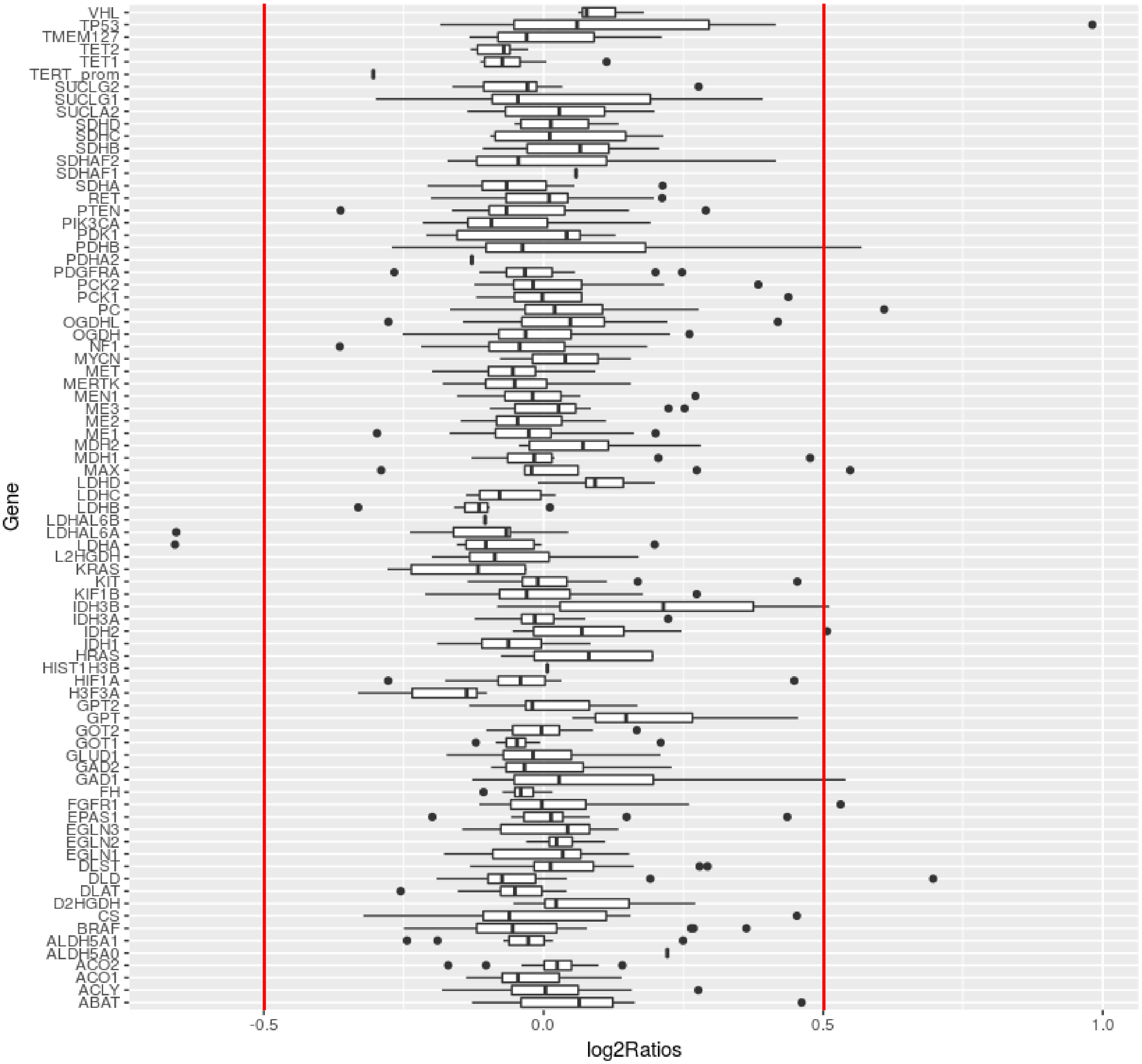

VHL
TP53
TMEM127
TET2
TET1
TERT_prom
SUCLG2
SUCLG1
SUCLA2
SDHD
SDHC
SDHB
SDHAF2
SDHAF1
SDHA
RET
PTEN
PIK3CA
PDK1
PDHB
PDHA2
PDGFRA
PCK2
PCK1
PC
OGDHL
OGDH
NF1
MYCN
MET
MERTK
MEN1
ME3
ME2
ME1
MDH2
MDH1
MAX
LDHD
LDHC
LDHB
LDHAL6B
LDHAL6A
LDHA
L2HGDH
KRAS
KIT
KIF1B
IDH3B
IDH3A
IDH2
IDH1
HRAS
HIST1H3B
HIF1A
H3F3A
GPT2
GPT
GOT2
GOT1
GLUD1
GAD2
GAD1
FH
FGFR1
EPAS1
EGLN3
EGLN2
EGLN1
DLST
DLD
DLAT
D2HGDH
CS
BRAF
ALDH5A1
ALDH5A0
ACO2
ACO1
ACLY
ABAT
Gene
-0.5
0.0
0.5
1.0
log2Ratios

Control 4

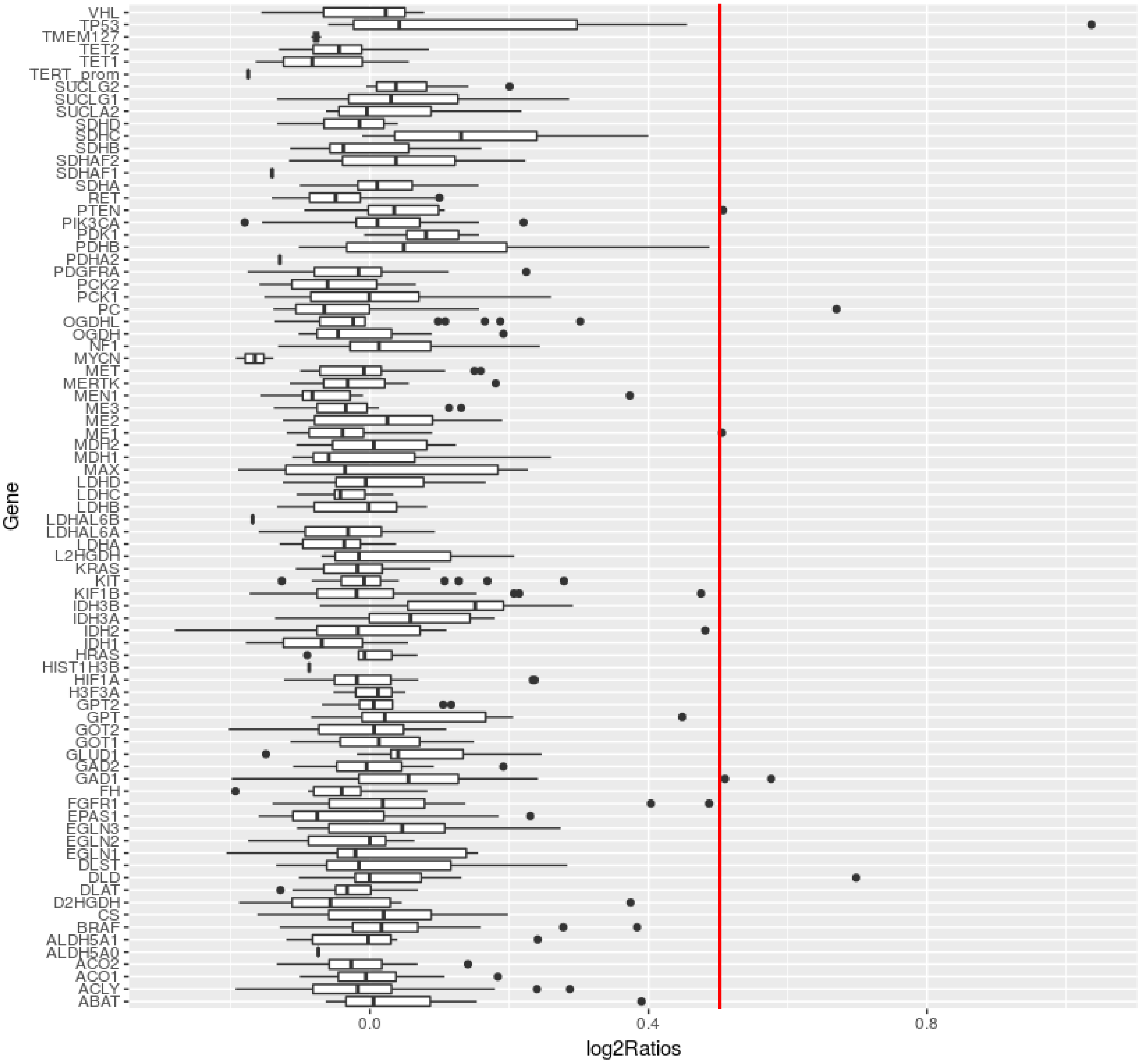

VHL
TP53
TMEM127
TET2
TET1
TERT_prom
SUCLG2
SUCLG1
SUCLA2
SDHD
SDHC
SDHB
SDHAF2
SDHAF1
SDHA
RET
PTEN
PIK3CA
PDK1
PDHB
PDHA2
PDGFRA
PCK2
PCK1
PC
OGDHL
OGDH
NF1
MYCN
MET
MERTK
MEN1
ME3
ME2
ME1
MDH2
MDH1
MAX
LDHD
LDHC
LDHB
LDHAL6B
LDHAL6A
LDHA
L2HGDH
KRAS
KIT
KIF1B
IDH3B
IDH3A
IDH2
IDH1
HRAS
HIST1H3B
HIF1A
H3F3A
GPT2
GPT
GOT2
GOT1
GLUD1
GAD2
GAD1
FH
FGFR1
EPAS1
EGLN3
EGLN2
EGLN1
DLST
DLD
DLAT
D2HGDH
CS
BRAF
ALDH5A1
ALDH5A0
ACO2
ACO1
ACLY
ABAT
Gene
0.0
0.4
0.8
log2Ratios

Control 5

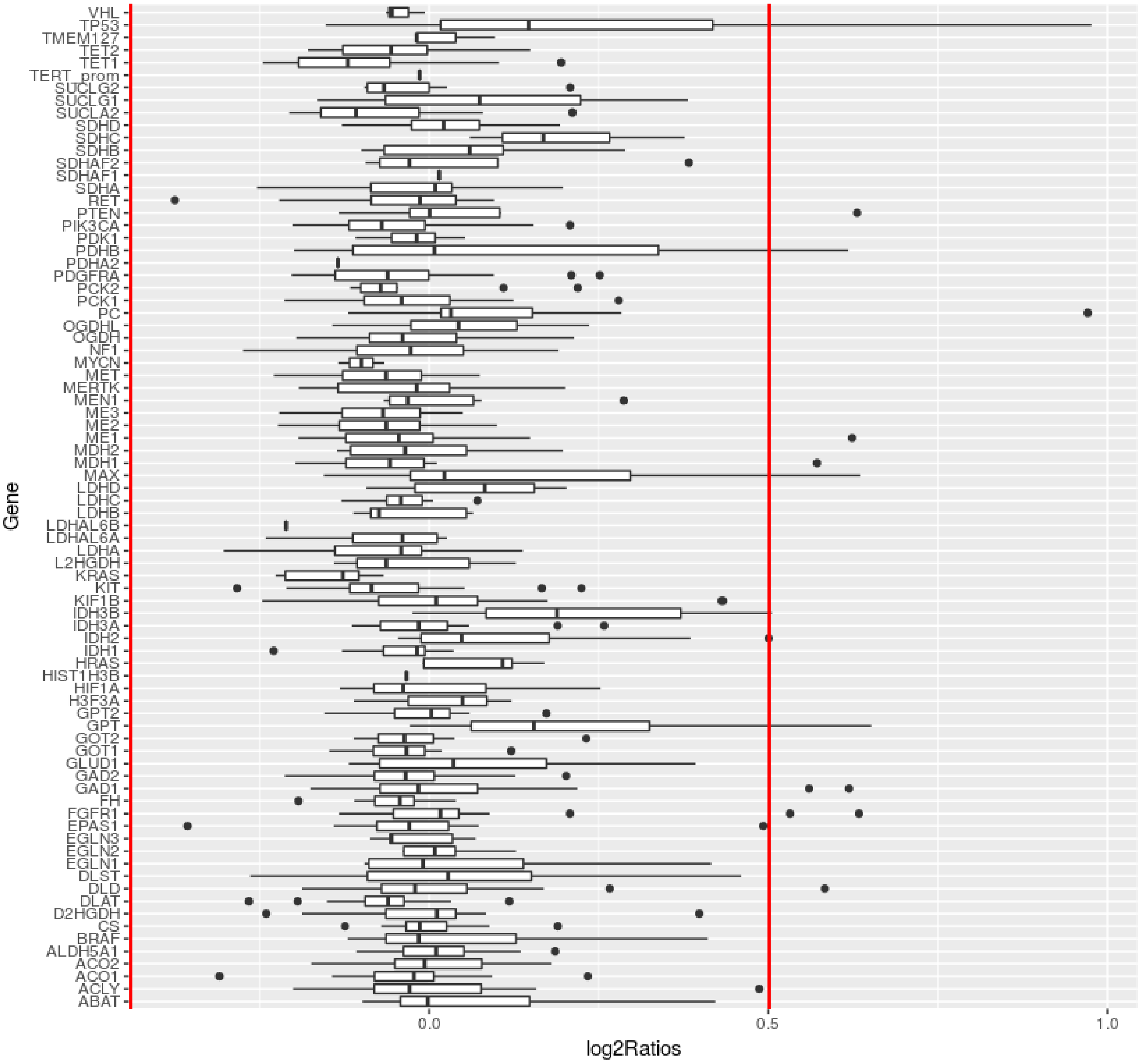
VHL
TP53
TMEM127
TET2
TET1
TERT_prom
SUCLG2
SUCLG1
SUCLA2
SDHD
SDHC
SDHB
SDHAF2
SDHAF1
SDHA
RET
PTEN
PIK3CA
PDK1
PDHB
PDHA2
PDGFRA
PCK2
PCK1
PC
OGDHL
OGDH
NF1
MYCN
MET
MERTK
MEN1
ME3
ME2
ME1
MDH2
MDH1
MAX
LDHD
LDHC
LDHB
LDHAL6B
LDHAL6A
LDHA
L2HGDH
KRAS
KIT
KIF1B
IDH3B
IDH3A
IDH2
IDH1
HRAS
HIST1H3B
HIF1A
H3F3A
GPT2
GPT
GOT2
GOT1
GLUD1
GAD2
GAD1
FH
FGFR1
EPAS1
EGLN3
EGLN2
EGLN1
DLST
DLD
DLAT
D2HGDH
CS
BRAF
ALDH5A1
ACO2
ACO1
ACLY
ABAT
Gene
0.0
0.5
1.0
log2Ratios

Control 6

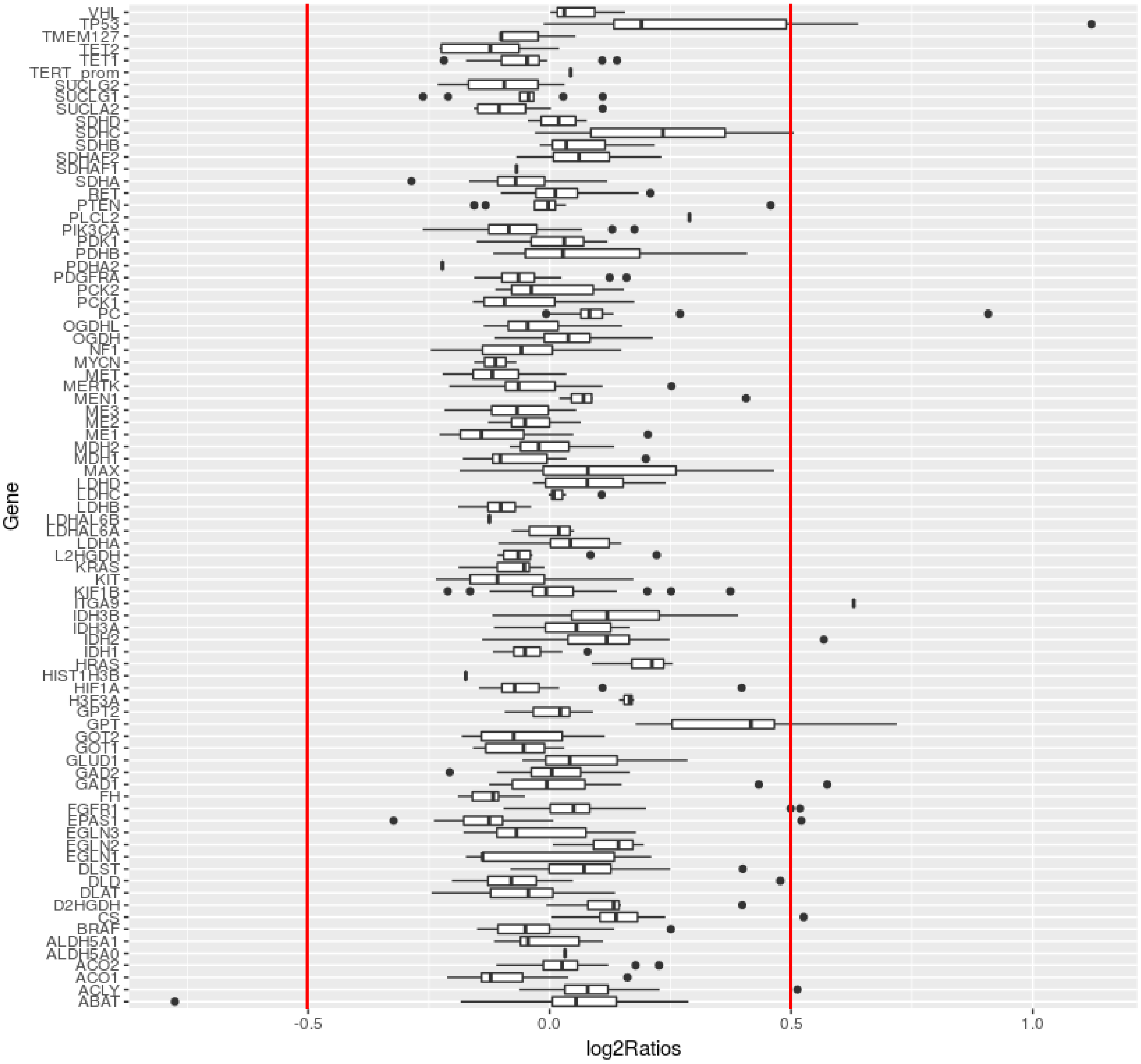
Gene
log2Ratios
-0.5
0.0
0.5
1.0
VHL
TP53
TMEM127
TET2
TET1
TERT_prom
SUCLG2
SUCLG1
SUCLA2
SDHD
SDHC
SDHB
SDHAF2
SDHAF1
SDHA
RET
PTEN
PLCL2
PIK3CA
PDK1
PDHB
PDHA2
PDGFRA
PCK2
PCK1
PC
OGDHL
OGDH
NF1
MYCN
MET
MERTK
MEN1
ME3
ME2
ME1
MDH2
MDH1
MAX
LDHD
LDHC
LDHB
LDHAL6B
LDHAL6A
LDHA
L2HGDH
KRAS
KIT
KIF1B
ITGA9
IDH3B
IDH3A
IDH2
IDH1
HRAS
HIST1H3B
HIF1A
H3F3A
GPT2
GPT
GOT2
GOT1
GLUD1
GAD2
GAD1
FH
FGFR1
EPAS1
EGLN3
EGLN2
EGLN1
DLST
DLD
DLAT
D2HGDH
CS
BRAF
ALDH5A1
ALDH5A0
ACO2
ACO1
ACLY
ABAT

Control 7

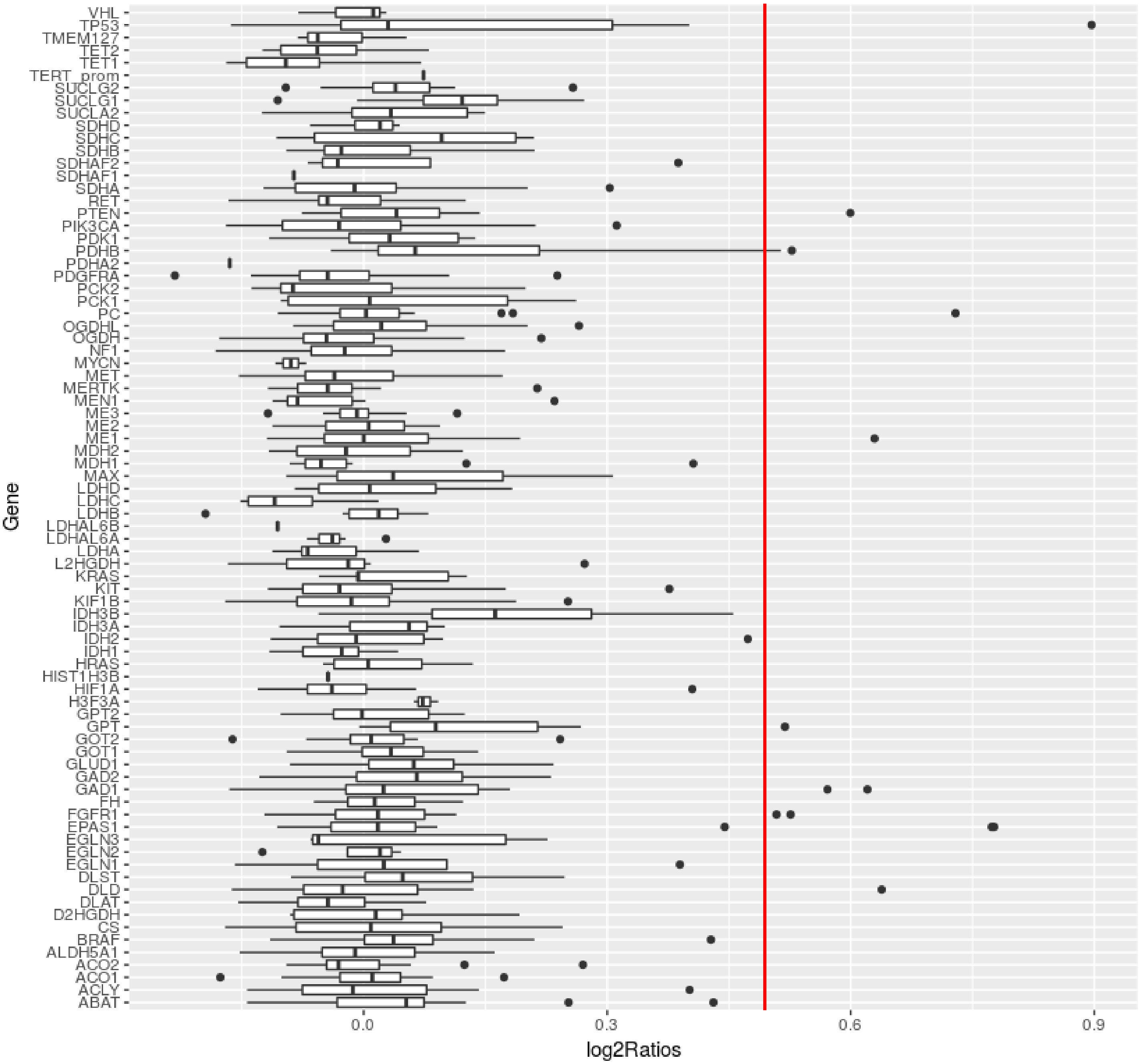

VHL
TP53
TMEM127
TET2
TET1
TERT_prom
SUCLG2
SUCLG1
SUCLA2
SDHD
SDHC
SDHB
SDHAF2
SDHAF1
SDHA
RET
PTEN
PIK3CA
PDK1
PDHB
PDHA2
PDGFRA
PCK2
PCK1
PC
OGDHL
OGDH
NF1
MYCN
MET
MERTK
MEN1
ME3
ME2
ME1
MDH2
MDH1
MAX
LDHD
LDHC
LDHB
LDHAL6B
LDHAL6A
LDHA
L2HGDH
KRAS
KIT
KIF1B
IDH3B
IDH3A
IDH2
IDH1
HRAS
HIST1H3B
HIF1A
H3F3A
GPT2
GPT
GOT2
GOT1
GLUD1
GAD2
GAD1
FH
FGFR1
EPAS1
EGLN3
EGLN2
EGLN1
DLST
DLD
DLAT
D2HGDH
CS
BRAF
ALDH5A1
ACO2
ACO1
ACLY
ABAT
Gene
0.0
0.3
0.6
0.9
log2Ratios

Control 8

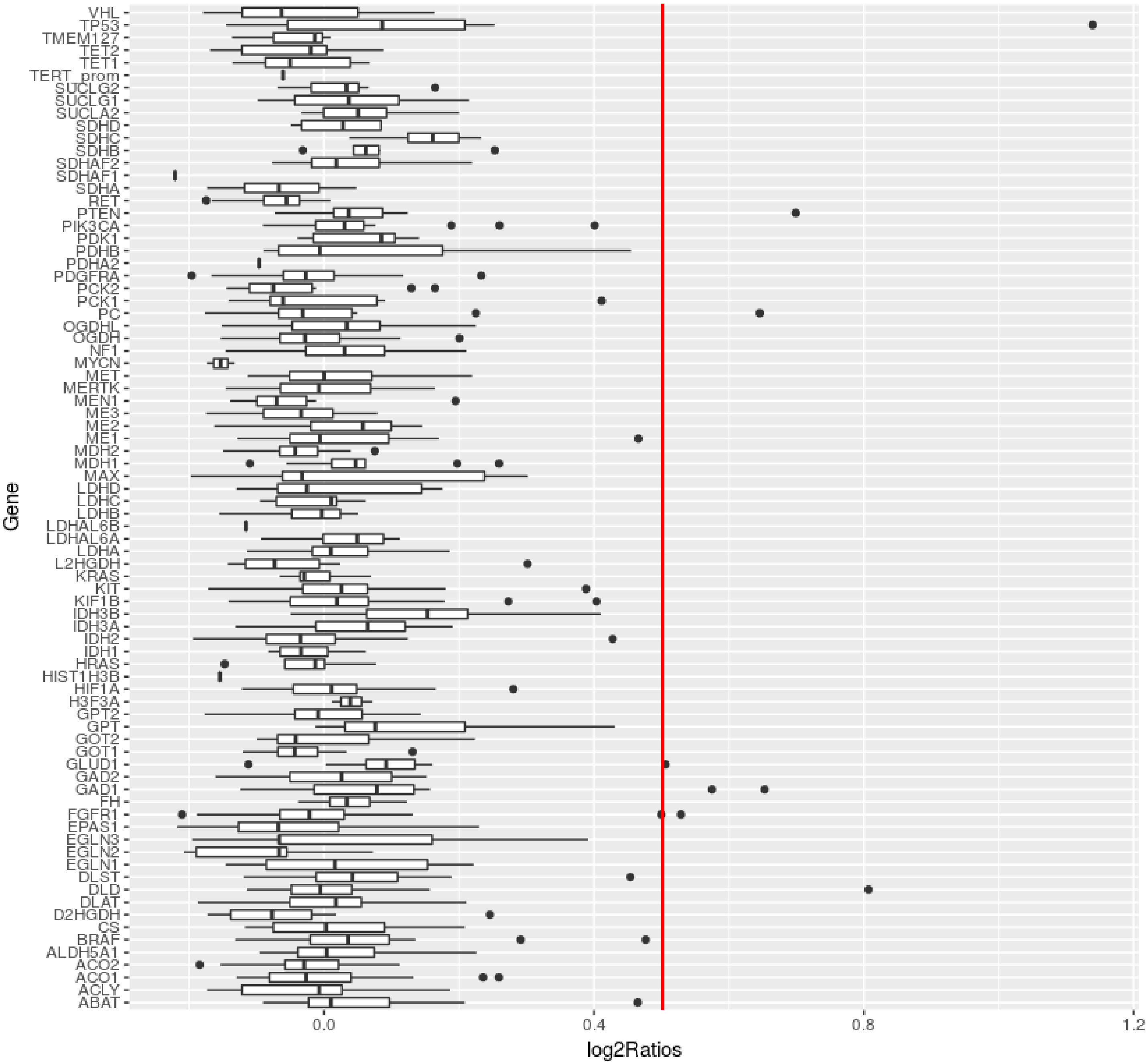
VHL
TP53
TMEM127
TET2
TET1
TERT_prom
SUCLG2
SUCLG1
SUCLA2
SDHD
SDHC
SDHB
SDHAF2
SDHAF1
SDHA
RET
PTEN
PIK3CA
PDK1
PDHB
PDHA2
PDGFRA
PCK2
PCK1
PC
OGDHL
OGDH
NF1
MYCN
MET
MERTK
MEN1
ME3
ME2
ME1
MDH2
MDH1
MAX
LDHD
LDHC
LDHB
LDHAL6B
LDHAL6A
LDHA
L2HGDH
KRAS
KIT
KIF1B
IDH3B
IDH3A
IDH2
IDH1
HRAS
HIST1H3B
HIF1A
H3F3A
GPT2
GPT
GOT2
GOT1
GLUD1
GAD2
GAD1
FH
FGFR1
EPAS1
EGLN3
EGLN2
EGLN1
DLST
DLD
DLAT
D2HGDH
CS
BRAF
ALDH5A1
ACO2
ACO1
ACLY
ABAT
Gene
0.0
0.4
0.8
1.2
log2Ratios

Control 9

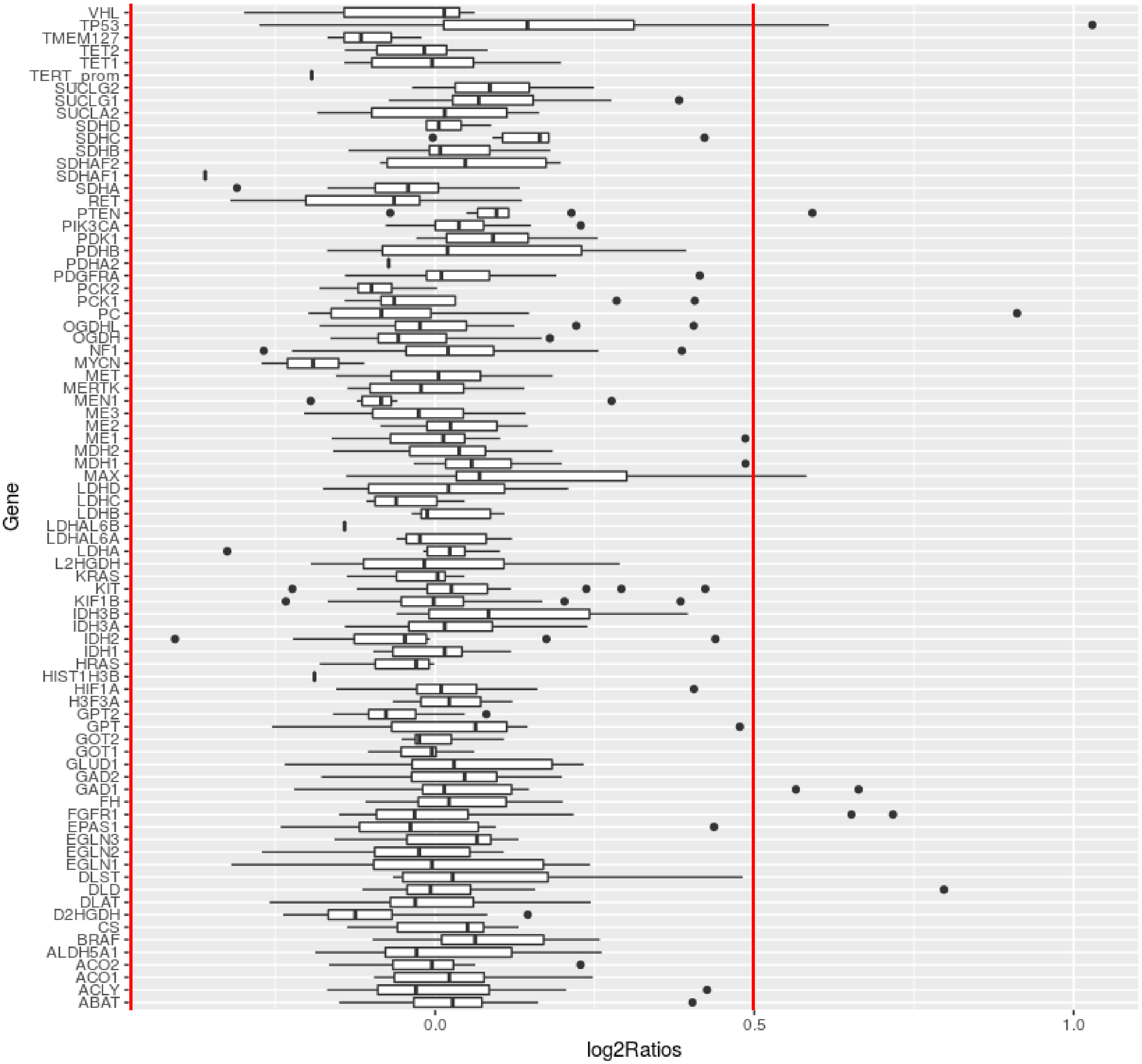
VHL
TP53
TMEM127
TET2
TET1
TERT_prom
SUCLG2
SUCLG1
SUCLA2
SDHD
SDHC
SDHB
SDHAF2
SDHAF1
SDHA
RET
PTEN
PIK3CA
PDK1
PDHB
PDHA2
PDGFRA
PCK2
PCK1
PC
OGDHL
OGDH
NF1
MYCN
MET
MERTK
MEN1
ME3
ME2
ME1
MDH2
MDH1
MAX
LDHD
LDHC
LDHB
LDHAL6B
LDHAL6A
LDHA
L2HGDH
KRAS
KIT
KIF1B
IDH3B
IDH3A
IDH2
IDH1
HRAS
HIST1H3B
HIF1A
H3F3A
GPT2
GPT
GOT2
GOT1
GLUD1
GAD2
GAD1
FH
FGFR1
EPAS1
EGLN3
EGLN2
EGLN1
DLST
DLD
DLAT
D2HGDH
CS
BRAF
ALDH5A1
ACO2
ACO1
ACLY
ABAT
Gene
0.0
0.5
1.0
log2Ratios

Control 10

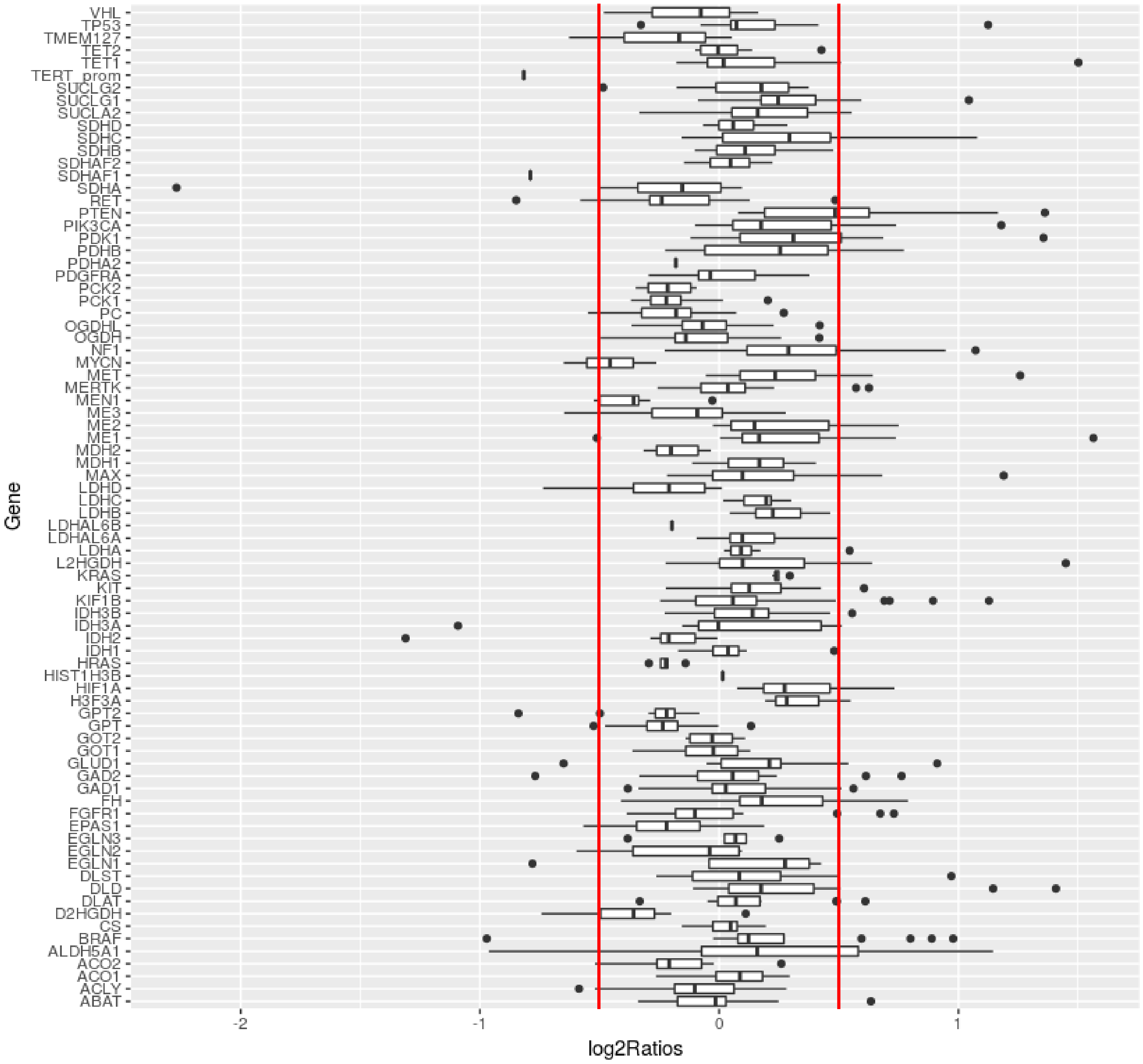
VHL
TP53
TMEM127
TET2
TET1
TERT_prom
SUCLG2
SUCLG1
SUCLA2
SDHD
SDHC
SDHB
SDHAF2
SDHAF1
SDHA
RET
PTEN
PIK3CA
PDK1
PDHB
PDHA2
PDGFRA
PCK2
PCK1
PC
OGDHL
OGDH
NF1
MYCN
MET
MERTK
MEN1
ME3
ME2
ME1
MDH2
MDH1
MAX
LDHD
LDHC
LDHB
LDHAL6B
LDHAL6A
LDHA
L2HGDH
KRAS
KIT
KIF1B
IDH3B
IDH3A
IDH2
IDH1
HRAS
HIST1H3B
HIF1A
H3F3A
GPT2
GPT
GOT2
GOT1
GLUD1
GAD2
GAD1
FH
FGFR1
EPAS1
EGLN3
EGLN2
EGLN1
DLST
DLD
DLAT
D2HGDH
CS
BRAF
ALDH5A1
ACO2
ACO1
ACLY
ABAT
Gene
-2
-1
0
1
log2Ratios
